# An expanded landscape of human long noncoding RNA

**DOI:** 10.1093/nar/gkz621

**Published:** 2019-07-27

**Authors:** Shuai Jiang, Si-Jin Cheng, Li-Chen Ren, Qian Wang, Yu-Jian Kang, Yang Ding, Mei Hou, Xiao-Xu Yang, Yuan Lin, Nan Liang, Ge Gao

**Affiliations:** Biomedical Pioneering Innovation Center (BIOPIC), Beijing Advanced Innovation Center for Genomics (ICG), Center for Bioinformatics (CBI), and State Key Laboratory of Protein and Plant Gene Research at School of Life Sciences, Peking University, Beijing 100871, China

## Abstract

Long noncoding RNAs (lncRNAs) are emerging as key regulators of multiple essential biological processes involved in physiology and pathology. By analyzing the largest compendium of 14,166 samples from normal and tumor tissues, we significantly expand the landscape of human long noncoding RNA with a high-quality atlas: RefLnc (**Ref**erence catalog of **Lnc**RNA). Powered by comprehensive annotation across multiple sources, RefLnc helps to pinpoint 275 novel intergenic lncRNAs correlated with sex, age or race as well as 369 novel ones associated with patient survival, clinical stage, tumor metastasis or recurrence. Integrated in a user-friendly online portal, the expanded catalog of human lncRNAs provides a valuable resource for investigating lncRNA function in both human biology and cancer development.

## INTRODUCTION

Long noncoding RNAs (lncRNAs) are defined as non-coding transcripts longer than 200 nt (1). They have been demonstrated to conduct diverse functions in multiple biological processes, including suppression of DNA synthesis (2), transcriptional regulation in *cis* or *trans* (3,4), post-transcriptional regulation of RNA (5,6) and regulation of protein translation (7), and they play important regulatory roles in various other physiological and pathological processes (8–12).

A high-quality and comprehensive lncRNA annotation is a cornerstone requirement of subsequent functional investigation. However, while tremendous efforts have been devoted to systematically characterizing lncRNAs in the human genome in recent years (13–15), large discrepancies still exist in the current major annotations. 23.4% of lncRNAs are found in only one gene model among GENCODE (13,16), RefSeq (14,17) and lncRNAdb (15,18), even with very loose criteria (Supplementary Figure S1A), which may be partly due to the relatively low and tissue-specific expression of lncRNAs (19,20).

Here, we use a compendium of 14,166 poly-A+ RNA-Seq libraries across 30 normal tissues, two cell lines and 18 tumors to comprehensively interrogate the physiological human poly-A+ transcriptome. In addition to verifying 50,380 known lncRNAs out of 51,834 lncRNAs, we have identified 27,520 novel lncRNA transcripts grouped in 20,518 gene loci over major references (see Materials and Methods for details). The information of all 77,900 lncRNAs, known and novel, is combined into a comprehensive human lncRNA database: RefLnc (**Ref**erence catalog of **Lnc**RNA). Using this valuable resource, we are able to identify hundreds of lncRNAs associated with various physiological traits and cancer progression. Both the assembly and the analysis results are publicly available through our interactive online portal at http://reflnc.gao-lab.org/.

## MATERIALS AND METHODS

### RNA-Seq datasets

We use two RNA-Seq datasets. For transcriptome reconstruction, we have screened 7,849 RNA-Seq samples in the GTEx project (v6) (Supplementary Table S1) based on three criteria: (i) normal human tissue/cell line (SMSTYP = ‘Normal’); (ii) RNA integrity number (RIN) value > 6.0; (iii) donors meeting the overall eligibility criteria for GTEx collection based on answers to eligibility questions (INCEXC = ‘TRUE’). For analysis in tumor, we filter out FFPE (formalin fixed paraffin embedded) samples from The Cancer Genome Atlas (TCGA) data and retain 6,317 samples from 18 tumors (Supplementary Table S2) that are frozen soon after surgery to prevent degradation of the RNA and DNA.

### High-performance computing

Computational analysis is performed using the high-performance computing platform of the Center for Life Sciences of Peking University.

### Reference gene model

We merge the lncRNA transcript models in major references including GENCODE v23, RefSeq (ftp://ftp.ncbi.nlm.nih.gov/genomes/Homo_sapiens/GFF/ref_GRCh38.p2_top_level.gff3) and lncRNAdb (downloaded on 27 July 2015) according to the following criteria. For multiple-exon transcripts, transcripts with the same sequence and matching splicing patterns are considered as redundant. For single-exon transcripts, transcripts with >80% sequence similarity are considered as redundant. Among the redundant transcripts, the ones annotated in GENCODE are retained. Annotations for other transcripts, such as those of protein-coding genes and pseudogenes, are retained from GENCODE. The final merged model consists of 79,795 protein-coding transcripts and 51,834 lncRNAs, which is used as a guided reference for read mapping and transcript assembly.

### Read mapping and transcriptome assembly

A standard RNA-Seq analysis pipeline is employed on all samples. We use HISAT2 (version 2.0.1-beta) (21) to map the sequencing reads to the human reference genome (version hg38/GRCh38) with the reference splice sites provided (–known-splicesite-infile). We use StringTie (v1.2.2) (22) to assemble transcripts in a reference-guided manner (-G). The reference and assembled transcript models are merged by StringTie merge (-F1) to obtain the merged transcript model. Novel transcripts are obtained by comparing the merged transcript model with the reference model by cuffcompare (23) (code! = ‘=’ && code! = ‘c’). The preliminary transcript model is obtained by merging the reference transcript model and novel transcript model directly.

### Estimating expression abundance and normalization

We estimate the expression levels (FPKM) and read coverage for the preliminary transcript model by running StringTie (v1.2.2) (22) in its expression abundance estimation mode (StringTie -e -b). Quantile normalization is applied to account for library size factors. Although quantile normalization was originally developed for microarrays, it has come to be widely used for normalization of RNA-Seq data (24–34), as well as cross-study/cross-platform normalization (27,35–39) to account for variations within and between datasets. Specifically, quantile normalization is employed in large-scale RNA-Seq samples of GTEx (24,25) and TCGA project (27,31,32) to eliminate the systematic difference. Moreover, studies indicate that quantile normalization shows similar results to other methods such as DESeq and TMM in aspect of high correlation between normalized counts and qRT-PCR data (absolute value or fold change) in real or simulated data (33,34).

### Transcript Confidence Score (TCS)

The Transcript Confidence Score indicates how well supported a transcript model is, based on the number of uniquely mapped junction reads, expression levels and sample recurrence. The Transcript Confidence Score is calculated according to the formula below:

\begin{equation*}{{\rm TCS}_t} = \frac{{100}}{3} \times \left( {\frac{{{J_t}}}{{\max J}} + \frac{{{E_t}}}{{\max E}} + \frac{{{R_t}}}{{\max R}}} \right)\end{equation*}

${J_t}$ is the average number of uniquely mapped junction reads of transcript *t* in the maximally expressed sample; ${E_t}$ is the 95th quantile expression (FPKM) of transcript *t;*${R_t}$ is the number of samples in which transcript *t* is expressed (FPKM > 0.1)

### Filtration of background noise

To define an optimal TCS cutoff for distinguishing *bona fide* transcripts from background, we calculate the TCS for the 46,519 verified multiple-exon known lncRNAs and 78,146 multiple-exon mRNAs as well as for randomly shuffled intergenic transcripts (Supplementary Figure S1B). The random intergenic transcripts are generated by bedtools shuffle (40) according to the structures of known mRNAs and lncRNAs (Supplementary Figure S1B). An optimal cutoff for distinguishing *bona fide* transcripts from background noise is the value for which the point on the ROC curve has the minimum distance to the upper left corner (where sensitivity = 1 and specificity = 1). By Pythagoras' theorem, this distance is sqrt((1 – sensitivity)² + (1 – specificity)²). Novel transcripts with TCS below this cutoff are excluded from the follow-up analysis.

### LncRNA identification and classification

We identify novel lncRNAs through the two following filters: (i) size selection (length > 200 bp) and (ii) lack of coding potential. We develop a stringent filtering pipeline aiming at removing novel transcripts with evidence for protein-coding potential. First, we integrate Coding Potential Calculator (CPC) (41) and Coding Potential Assessment Tool (CPAT) (42): transcripts that are predicted to lack coding potential by either CPAT or CPC are regarded as preliminary noncoding RNAs. Second, we make conceptual translations for three frames of these preliminary noncoding RNAs by ORFfinder (https://www.ncbi.nlm.nih.gov/orffinder/). Finally, we scan these translated sequences in the Pfam (43) database with three cutoffs (ga/nc/tc), in the 2,201 mass spectrometry samples from Human Proteome Map (44) (by X!tandem (45)) and in the 61 Ribo-Seq profiling samples (by RibORF (46)) from SRA database (47) (Supplementary Table S3). We remove the transcripts with any hit in the Pfam database, the mass spectrometry data or the Ribo-Seq samples, and obtain the final lncRNA catalog.

As for classification, lncRNAs are compared to protein-coding transcripts by cuffcompare (23), and lncRNAs with the code ‘u’ are defined as ‘intergenic’. The lncRNAs overlapping with the exons of protein-coding transcripts in the same strand are defined as ‘sense’. The lncRNAs transcribed from the antisense strand of protein-coding genes are classified as ‘antisense’. The remaining lncRNAs are referred to as ‘others’.

### Validation of novel lncRNA transcripts by quantitative RT-PCR and Sanger sequencing

We select 100 novel intergenic lncRNAs (42 single-exon and 58 multi-exon) for biological validation according to the following criteria: (i) not overlap with any annotated transcripts; (ii) with top expression (FPKM > 1) in either H1-ESC, HepG2, HelaS3 or K562 based on the expression levels assayed by RNA-Seq of cell lines from ENCODE (http://genome.ucsc.edu/cgi-bin/hgTrackUi?db=hg38&c=chr7&g=wgEncodeRegTxn); (iii) blat (48) these transcripts to hg38 genome, and remove the transcripts mapped to multiple positions of genome with Coverage ≥90% and Identity ≥90%, and only retain transcripts that can be mapped to genome with 100% coverage and 100% identity.

Primer pairs are designed using the Primer-Premier 5 (Premier Biosoft Interpairs, Palo Alto, CA, USA) and are mapped against the human genome (hg38) by UCSC In-Silico PCR (49) to ensure specificity. Unique primer pairs are designed for 93 lncRNAs. As for each multi-exon transcript, there are primers designed to span exon junctions.

RNA is isolated from H1-ESC, HepG2, HelaS3 and K562 cells in Trizol (Invitrogen) respectively. 1–5 ug RNA is converted into cDNA using random primers and the HiScript^®^ II 1st Strand cDNA Synthesis Kit (+gDNA wiper) (Vazyme). Quantitative real-time PCR (qPCR) is performed using ChamQTM Universal SYBR^®^ qPCR Master Mix (Vazyme) on Roche480 Real-Time PCR System for each transcript in the cell line with the highest expression level in RNA-Seq data. Housekeeping gene, actin, is used as positive control. Data is normalized by housekeeping gene using the delta Ct method. The amplicons are further analyzed by Sanger sequencing.

### Tissue specificity

To evaluate the tissue specificity of the transcripts, we apply the previously defined entropy-based ‘tissue specificity score’, which relies on Jensen–Shannon (JS) divergence to quantify the similarity between the transcript expression patterns and another pre-defined pattern in which the transcript is expressed in only one tissue (19). A higher tissue specificity score represents higher tissue specificity of a transcript. According to the previous study (19), the JS divergence of two probability distributions *p*^1^ and *p*^2^ is defined to be

\begin{equation*}JS\ \left( {{p^1},{p^2}} \right) = \ H\left( {\frac{{{p^1} + {p^2}}}{2}} \right) - \ \frac{{H\left( {{p^1}} \right) + H\left( {{p^2}} \right)}}{2},\end{equation*}

where *H* is the entropy of a probability distribution:

\begin{equation*}p\ = \left( {{p_1},{p_2}, \ldots ,{p_n}} \right)\ ,0 \le {p_i} \le 1\ {\rm{and\ \ }}\mathop \sum \limits_{i\ = \ 1}^n {p_i} = \ 1\end{equation*}

\begin{equation*}H\ \left( p \right) = {\rm{\ }} - \mathop \sum \nolimits_{i\ = \ 1}^n {p_i}{\rm{log}}\left( {{p_i}} \right)\end{equation*}

The distance between two tissue expression patterns ${e^1}\ {\rm{and}}\ {e^2},\ \ {e^i} = \ ( {e_1^i,e_2^i,..,e_n^i} )$ is

\begin{equation*}J{S_{dist}}\ \left( {{e^1},{e^2}} \right) = \sqrt {JS\left( {{e^1},{e^2}} \right)} \ \end{equation*}

The tissue specificity of a transcript's expression pattern, *e*, across *n* tissues with respect to tissue *t* is defined as

\begin{equation*}J{S_{sp}}\ \left( {e{\rm{|}}t} \right) = \ 1 - J{S_{dist}}\left( {e,{e^t}} \right)\end{equation*}

where *e^t^* is a pre-defined expression pattern that represents the extreme case in which a transcript is expressed in only one tissue *t*.

\begin{equation*}{e^t} = \left( {e_1^t,e_2^t, \ldots ,e_n^t} \right){\rm{\ }},{\rm s.t.}{\rm{\ \ }}e_i^t = \left\{ {\begin{array}{@{}*{1}{c}@{}} {1\ if\ i = t}\\ {\ 0\ {\rm otherwise}} \end{array}} \right.{\rm{\ }}\end{equation*}

Finally, the tissue specificity score of a transcript is defined as the maximal tissue specificity score across all n tissues of the transcripts expression pattern e:

\begin{equation*}{JS_{sp}}\ \left( e \right) = argma{x_t}\ J{S_{sp}}\left( {e{\rm{|}}t} \right),\ t\ = \ 1,2, \ldots ,n\end{equation*}

The expression pattern, e, is normalized as follows:

\begin{equation*}{{E^{\prime}\ }} = {\rm{\ }}\frac{{{\log_2}\left( {E + 1} \right)}}{{\mathop \sum \nolimits_{i\ = \ 1}^n {\log_2}\left( {{e_i} + 1} \right)}}\end{equation*}

### Splicing efficiency

We estimate splicing efficiency according to the method described previously (50). When calculating the expression of transcripts, we use a modified annotation containing an additional isoform per gene that spanned the whole gene locus. The splicing efficiency of a gene is calculated as the sum of the abundances of all originally annotated isoforms of the gene divided by the sum of the abundances of all isoforms including the spanning one.

\begin{eqnarray*}&&{\rm{splicing\ efficiency\ }} \nonumber\\\ &&= \frac{{{\rm{Abundance\ isoform}}1 + \ldots + {\rm{Abundance\ isoform\ n}}}}{{{\rm{Abundance\ isoform}}1 + \ldots + {\rm{Abundance\ isoform\ n}} + {\rm{Abundance\ spanning\ unspliced\ isoform}}}}\end{eqnarray*}

A higher splicing efficiency score represents higher splicing efficiency of a gene.

### Conservation analysis

The evolutionary conservation of the transcripts in our assembly is evaluated by the PhastCons score of 100 vertebrates downloaded from UCSC (http://hgdownload.soe.ucsc.edu/goldenPath/hg38/phastCons100way/hg38.phastCons100way.bw). We extract the scores from the genomic regions using bedtools (40) and divide the conservation scores by the transcript length.

### Inter-individual expression variability analysis

Inter-individual expression variability is estimated by normalizing the standard deviation to the average expression among donors for 23 normal tissues and two cell lines from both genders. The donors are selected by choosing all the female donors and randomly selecting an equal number of male donors. Transcripts from chromosomes X and Y are discarded and only transcripts expressed in the given tissue in at least one donor (FPKM > 0.1) are displayed.

### GWAS analysis

A list of 29,929 unique GWAS SNPs is obtained from the National Human Genome Research Institute's GWAS catalog (51) (accessed 15 March 2017). We focus on 3,425 reported intergenic and significant SNPs (*P*-value < 5e–8). The number of overlap between SNPs and the whole transcript locus (including the introns) is counted.

### Novel lincRNAs for functional screening

We focus on 7,143 novel multiple-exon intergenic lncRNAs with moderate-to-high expression (Q3 +1.5*IQR > 0.1 FPKM in at least one normal tissue, which is a more stringent cutoff than the general cutoff of maximum expression (>0.1 FPKM in at least one tissue)) for the functional screening of novel lincRNAs.

### Sex, race and age differential transcript expression analysis

We apply a linear mixed model (LMM) incorporating sex, race and age as covariates together with individual and tissue to investigate their effects on transcript expression. We consider individuals as block random effects and we use the function *lme* of the nlme package of R. The model is written as lme(fixed = Expression ∼ Tissue + Sex + Race + Age, random = ∼1|Individual). We only use samples in GTEx in this part of analysis.

### Tissue-specific expression patterns of race-biased novel lincRNAs

We apply the estimated odds ratio (OR) in Fisher's exact test (one-tailed test) to measure the tissue-specific expression pattern for race-biased novel lincRNAs.

\begin{equation*}\begin{array}{@{}*{1}{l}@{}} {{\rm{Odds\ ratio}}}\\ { = \frac{{{\rm{the\ number\ of\ race}}\_{\rm{biased\ transcripts\ specifically\ expressed\ in\ tissue\ }}t}}{{{\rm{the\ number\ of\ other\ race}}\_{\rm{biased\ transcripts}}}}/}\\ {\frac{{{\rm{the\ number\ of\ non}}\_{\rm{race}}\_{\rm{biased\ transcripts\ specifically\ expressed\ in\ tissue\ }}t}}{{{\rm{the\ number\ of\ other\ non}}\_{\rm{race}}\_{\rm{biased\ transcripts}}}}} \end{array}\end{equation*}

Therefore, an odds ratio >1 indicates that race-biased transcripts are enriched in tissue *t* compared to non-race-biased transcripts. Tissue-specific transcripts are defined as transcripts with tissue specificity score >0.6.

### Discovery of differentially expressed transcripts between tumor and normal tissues

We use the linear mixed model (LMM) for each tissue to investigate the effect of tumor/normal type on the transcript expression, incorporating sex, race and age as covariates together with individual as the random effects. Fold-change is calculated based on the ratio of the average expression levels in tumor and normal tissues. Transcripts that are significantly associated with tumor/normal type (FDR < 0.05) and with fold-change >1.5 are defined as differentially expressed transcripts between tumor and normal tissues. When we perform the differentially expression analysis, we’ve used the quantile normalization for the expression profiles of GTEx and TCGA samples to remove the batch effect (52).

### Identifying lncRNAs associated with tumor metastasis and recurrence

We investigate the effect of clinical outcomes on transcript expression by extending the LMM for each tumor to incorporate sex, race and age as covariates together with individual as the random effects. Fold-change is calculated based on the ratio of the average expression level between the two conditions. Transcripts that are significantly associated with clinical outcomes (FDR < 0.05), with fold-change >1.5, and moderately to highly expressed in the corresponding tumor (Q3 +1.5*IQR > 0.1 FPKM) are retained.

### Identifying lncRNAs associated with clinical stage

We investigate the effect of clinical stage on transcript expression by extending the LMM for each tumor to incorporate sex, race and age as covariates together with individual as the random effects. Transcripts that are significantly associated with clinical stage (FDR < 0.05) and moderately to highly expressed in the corresponding tumor (Q3 +1.5*IQR > 0.1 FPKM) are retained.

### Identifying lncRNAs associated with overall survival

We perform a multivariate Cox proportional hazard (Cox regression) analysis for each tumor (retaining only one sample of each individual) to assess the association between individual lncRNA expression and survival in the presence of race, age and sex as confounding factors. In addition, we conduct a survival analysis for each status, including clinical stage and mutation status (*EGFR, EML4* and *KRAS* in lung tumor). The hazard ratios (HRs) from the multivariate Cox regression analysis are used to identify protective (HR < 1) and risky lncRNAs (HR > 1). We use the cox.zph function to test the proportional-hazards assumption for each covariate, and there is strong evidence of non-proportional hazards for age in brain and ovary tumors. We accommodate the non-proportional hazards by dividing the age into strata to incorporate an interaction between age and time into the Cox regression model. Kaplan–Meier analysis with log-rank test is performed for specific cases, and the cutoff distinguishing the two groups is the median expression of patients with available survival information. The transcripts that are significantly associated with survival (FDR < 0.05) and moderately to highly expressed in the corresponding tumor (Q3 +1.5*IQR > 0.1 FPKM) are retained.

Given the relatively low expression of lncRNAs (19,20), the 0.1 FPKM is a widely used cutoff for filtering the expressed lncRNAs (53–55). Meanwhile, we’ve added another set of cancer-related lncRNAs with more stringent expression criterion (FPKM > 1) as Supplementary Table S4–S7.

### Comparisons of RefLnc with the other recent lncRNA catalogs

We compare the lncRNA transcripts in RefLnc with those in GENCODE v29, RefSeq (NCBI Homo sapiens Annotation Release 109), lncRNAdb v2, NONCODE v5, MiTranscriptome v2, CHESS v2 as well as FANTOM CAT (56) (FANTOM_CAT.lv3_robust.only_lncRNA.gtf) with the following criterion: the overlap of 1 bp in the exon (ignoring strand) is considered as redundant. The transcript assemblies of MiTranscriptome and FANTOM CAT are converted from GRCh37 to hg38 by UCSC liftOver (49).

### The independent datasets of normal human tissues and cancer cell lines

For the independent evaluation of transcript expression, we screen for two independent datasets of 1,131 samples of human normal tissues in the SRA database (Supplementary Table S8) and 935 samples of human cancer cell lines in the CCLE database (Supplementary Table S9). We select the normal samples from SRA based on the following criteria: (i) sample-type confidence > 0.7; (ii) filter out samples with ontology of description of ‘cell line’, ‘disease’ and ‘cancer’; (iii) paired-end sequencing. When we evaluate the coverage of the novel lncRNAs in the SRA samples, the highly expressed novel lncRNAs in GTEx tissues contained in the SRA are novel lncRNAs with expression (Q3 +1.5*IQR) higher than 1 FPKM.

### The set of putatively functional lncRNAs

The lncRNAs are considered putatively functional if they are associated with any of the following traits: (i) overlap significant trait-associated SNPs located within intergenic regions; (ii) be remarkably differentially expressed between tumor and normal tissues; (iii) be significantly correlated with physiological traits (sex/age/race); (iv) be significantly associated with survival, metastasis, clinical stage or tumor recurrence.

### Statistics analysis

We adjust the false discovery rate (FDR) using the Benjamini-Hochberg procedure. All the statistical methods are performed by the computing environment R. Statistics are done using R 3.2.4 (57), the data.table (58) the preprocessCore (59), the plyr (60), the nlme (61), the stringr (62) and the survival (63,64) packages.

## RESULTS

### An expanded landscape of human lncRNAs

**Figure 1.**
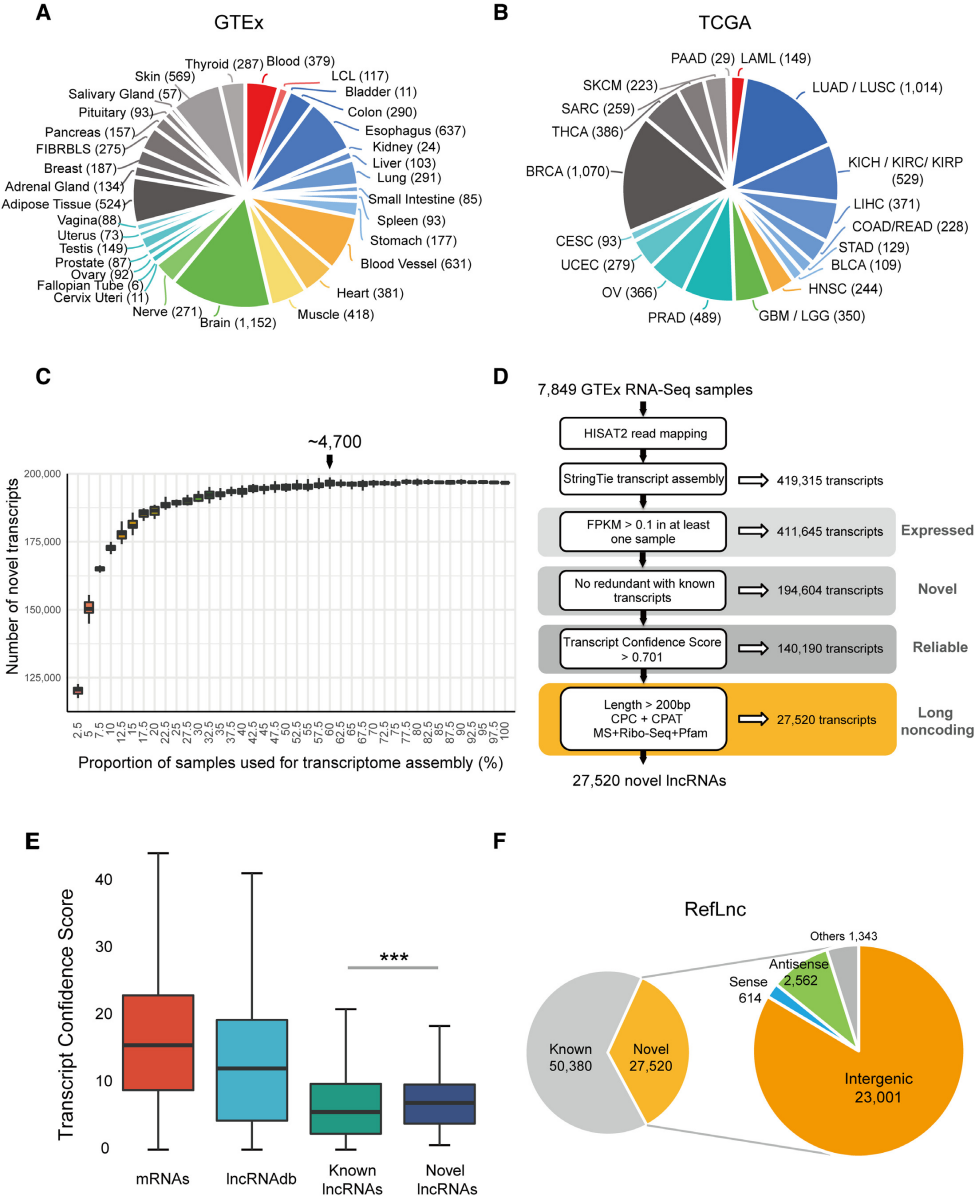
Reference-guided transcriptome assembly greatly expands the landscape of human lncRNAs. (**A**) The composition of the 7,849 physiological samples of 30 physiological tissues and two cell lines used for transcriptome reconstruction. (**B**) The composition of the 6,317 samples of 18 tumors from TCGA. (**C**) The number of novel transcripts assembled using different size of sample sets. We assemble the transcriptome by analyzing 40 datasets of RNA-Seq samples. Each dataset contains a different number of samples, from 196 to 7,849 samples (rising by 2.5% of the whole dataset). In each dataset, samples are randomly selected by 20 times according to the original tissue proportion of the whole dataset, which is shown in each boxplot. In addition, each dataset includes all type of sexes and races. (**D**) An integrative computational pipeline for lncRNA identification. The sequencing reads are mapped to the human reference genome (version hg38/GRCh38) and reference-guided transcriptome assembly is carried out on each RNA-Seq library. The resulting assembled transcript models for each library are merged to a consensus transcript assembly and filtered to obtain the reliably expressed novel transcripts. Finally, novel lncRNAs are identified by two filters: (1) lack of coding potential; (2) size selection. (**E**) The Transcript Confidence Score (TCS) of novel lncRNAs is higher than that of known lncRNAs. *P*-values are calculated using the Wilcoxon rank sum test. ‘***’: *P*-value < 0.001. (**F**) In total, RefLnc contains 77,900 lncRNAs including the verified known and novel lncRNAs, and 83.6% of the novel lncRNAs are in intergenic regions.

To characterize the landscape of human lncRNAs across different tissues, cell lines and individuals, we interrogate the human transcriptome with 14,166 poly-A+ RNA-Seq libraries, including 7,849 from the Genotype-Tissue Expression (GTEx) project (24,65) and 6,317 from The Cancer Genome Atlas (TCGA) (66) (Figure 1A-B and see Materials and Methods for details). The dataset represents wide coverage of the human transcriptome, including 30 normal tissues, two cell lines and 18 tumors.

After mapping the reads of RNA-Seq libraries to the reference genome (hg38/GRCh38, see Methods for details), we verify 50,380 known lncRNAs out of 51,834 lncRNAs from GENCODE (v23), RefSeq and lncRNAdb. In specific, >95% of the known lncRNAs in GENCODE (96.9%, 26,966/27,841), RefSeq (97.6%, 24,714/25,314) and lncRNAdb (95.2%, 139/146) can be verified in at least one sample with >2x per-base coverage on average (67). This result confirms the generally high quality of the existing major annotations.

Given the significant genetic alterations in tumor cells (68), we assemble the transcripts only from physiological human samples. We curate 7,849 high-quality poly-A+ RNA-Seq libraries from 533 individuals, covering 30 physiological tissues and two cell lines (Epstein-Barr virus–transformed lymphocytes (LCL) and cultured fibroblasts from skin (FIBRBLS)), containing approximately 350 billion sequencing reads (Figure 1A). Employing a reference-guided assembly approach for cohorts of various sizes (see Methods for details), we find that the number of novel assembled transcripts exhibits a 1.24-fold increase when the sample size increases from ∼200 samples to ∼400 samples (Figure 1C). When the number of samples reaches ∼4,700, the number of novel transcripts approaches saturation (Figure 1C). From all 7,849 physiological samples, we obtain a human transcriptome with 411,645 primary expressed transcripts (FPKM > 0.1 in at least one sample) from 123,493 genes, nearly half of which (47.3%; 194,604) are novel (Figure 1D and see Methods for details). Our curated data provides a rich resource for the genome-wide exploration of novel transcripts.

We design a Transcript Confidence Score (TCS) to measure transcript quality based on uniquely mapped junction reads, expression levels and recurrence (Figure 1E and see Materials and Methods for details). ROC analysis indicates that TCS performs well, with an AUC of 0.961, and an optimal cutoff (0.701) of TCS is determined with high specificity (0.961) and sensitivity (0.918) (Supplementary Figure S1C). Thus, 140,190 reliable novel transcripts with TCS above 0.701 are retained for the follow-up analysis (Figure 1D). Among them, 92.9% are expressed in more than two different tissues, and 98.2% are detected in more than three samples (Supplementary Figure S1D–E). From the 140,190 novel transcripts, we further identify 27,520 novel lncRNAs at 20,518 loci by screening transcripts with lack of coding potential and transcript length longer than 200 bp (Figure 1D and see Materials and Methods for details). Most of these novel lncRNAs (83.6%, 23,001/27,520) are intergenic (Figure 1F), and more than half (52.9%, 14,551/27,520) have multiple exons. In addition, 89.6% of these novel lncRNAs are transcribed in more than two different tissues, and 96.6% could be reproducibly detected in more than three samples (Supplementary Figure S1D-E). We combine the novel lncRNAs with verified known ones into a comprehensive human lncRNA catalog (RefLnc), in which 35.3% (27,520/77,900) lncRNAs are novel (Figure 1F).

To further assess the robustness of these identified novel lncRNAs, we choose 100 novel intergenic lncRNAs (58 multi-exon and 42 single-exon) which are not overlapped with any annotated transcripts for quantitative RT-PCR (qRT-PCR) validation. Primer pairs are designed using the Primer-Premier 5 (Premier Biosoft Interpairs, Palo Alto, CA, USA) and mapped against the human genome (hg38) by UCSC In-Silico PCR (49) to ensure specificity. Unique primer pairs meeting these criteria are designed successfully for 93 lncRNAs (57 multi-exon and 36 single-exon, see Methods for details). Out of these 93 cases, 91.4% (52 multi-exon and 33 single-exon) are successfully validated by Sanger sequencing independently (Supplementary Table S10, Supplementary Figure S1F-I and see Materials and Methods for details). Meanwhile, we also find a significant correlation between qRT-PCR quantitation and RNA-Seq expression profiles (*P*-value = 6.95e–7, Spearman rho = 0.437).

### The characterization of human lncRNAs

**Figure 2.**
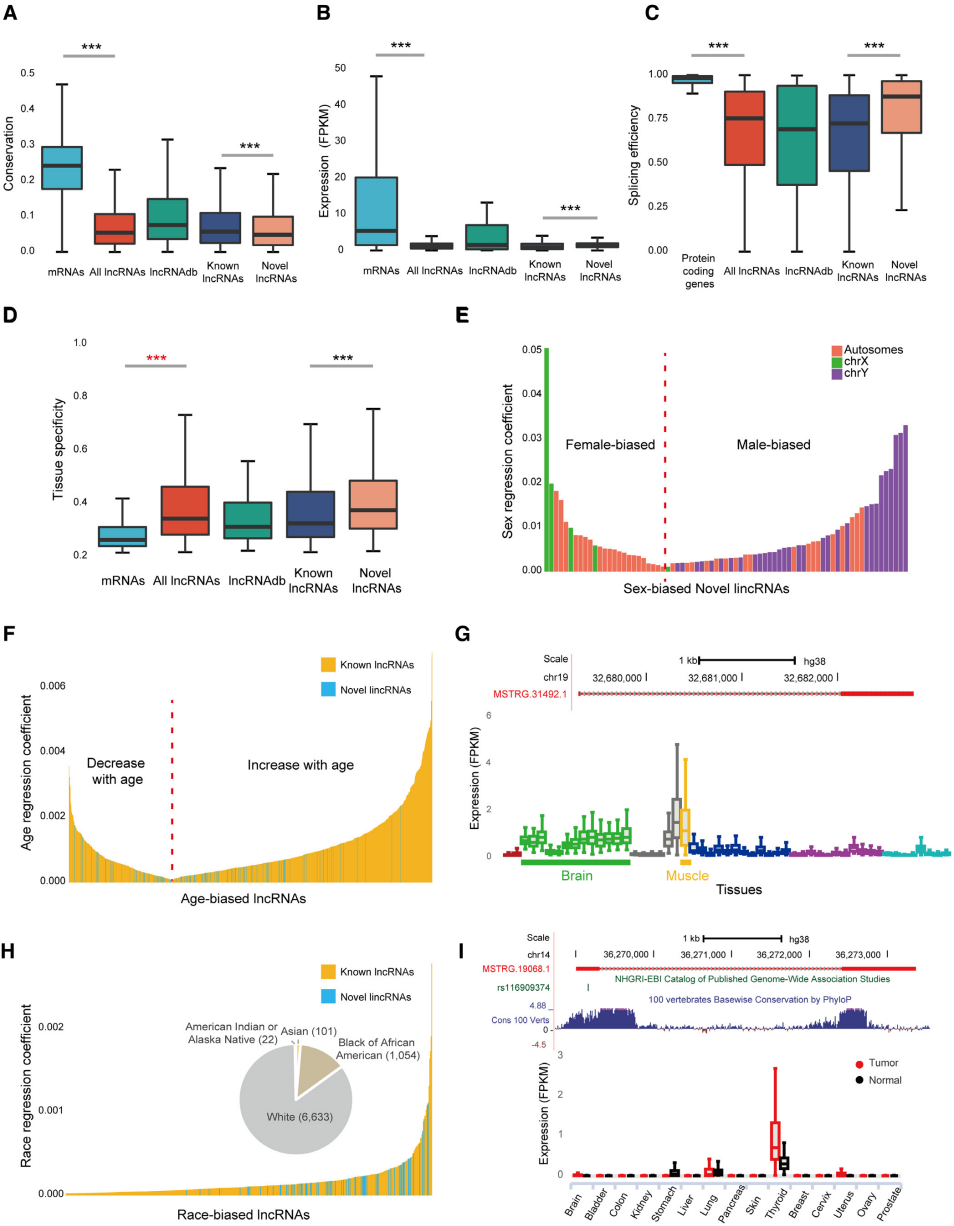
Characterization of the RefLnc assembly. (**A**) The conservation of lncRNAs is lower than that of mRNAs. (**B**) The expression levels of lncRNAs are lower than that of mRNAs, with 4.8-fold and 13.3-fold lower for median and mean expression levels, respectively. (**C**) lncRNAs have lower splicing efficiency than protein-coding genes. (**D**) lncRNAs are expressed in a much more tissue-specific manner than mRNAs. *P*-values are calculated using the Wilcoxon rank sum test. ‘***’: *P*-value < 0.001. (**E**) Sex-biased novel lincRNAs that are differentially expressed between males and females (FDR < 0.05). The transcripts on the left side of the red dotted line represent novel lincRNAs up-regulated in the female, while the right side represents novel lincRNAs up-regulated in the male. (**F**) Novel lincRNAs and known lncRNAs correlated with age (FDR < 0.001). The left side of the red dotted line indicates that the expression of the lncRNAs decreases with increasing age, while the right side indicates that the expression of the lncRNAs increases with the increasing age. (**G**) The genomic view and expression patterns in normal samples of the age-associated novel lincRNA MSTRG.31492.1. (**H**) Novel lincRNAs and known lncRNAs that are differentially expressed across different races (FDR < 0.05). The pie chart shows the population of the samples. (**I**) The genomic view and differential expression patterns between tumors and normal tissues of the novel lincRNA MSTRG.19068.1, which overlaps a thyroid cancer risk-associated SNP.

While lncRNAs and mRNAs share similar biogenesis pathways (69), they differ considerably in many aspects (50). Consistent with previous reports (19,20,50), lncRNA transcripts are shorter (Supplementary Figure S2A), with fewer exons (Supplementary Figure S2C) and lower GC content (Supplementary Figure S2E), and are less evolutionarily conserved (Figure 2A) than mRNAs. In addition, we find that lncRNAs have lower expression (Figure 2B) and less alternative splicing efficiency than protein-coding genes (Figure 2C, 14.0% of lncRNA genes are alternatively spliced while 85.5% of protein-coding genes are spliced), and lncRNAs are expressed in a much more tissue-specific manner than mRNAs (Figure 2D). The above results are still significant when the expression levels are controlled (Supplementary Figures S2B, D, F, S3A–C).

It has been proposed that the high correlation of transcriptional activity between neighboring noncoding and coding loci is an evidence for a *cis*-regulatory role of lncRNAs (70,71), while the co-expression between a lncRNA and its protein-coding neighbor may also result from proximal transcriptional activity in the surrounding open chromatin (72,73). We find that the correlation between neighboring lncRNA and mRNA pairs is significantly higher than the correlation between random neighbor pairs with the same distances (*P*-value < 2.2e–16, Wilcoxon test; Supplementary Figure S3D), which supports the *cis*-effect model.

### Multiple novel lincRNAs show sex/age/race-biased expression and overlap with trait-associated SNPs

Long intergenic non-coding RNAs (lincRNAs) are long non-coding RNAs that do not overlap annotated protein-coding genes. The lincRNAs such as XIST (74) and HOTAIR (75) have been functionally characterized in diverse gene regulation processes, organisms and human diseases. To identify potentially functional novel lincRNAs, we focus on 7,143 novel multiple-exon lincRNAs with moderate-to-high expression in the next sections (see Methods for details).

A mixed model with sex, age and race incorporated as covariates is employed to identify lncRNAs associated with these traits (see Methods for details). As a result, we detect 75 novel lincRNAs with strongly sex-biased expression patterns (false discovery rate (FDR) < 0.05, Figure 2E, Supplementary Table S11). Among them, 50 (66.7%) are male-biased and 25 are female-biased (Figure 2E). In addition to several known X inactivation lncRNAs like XIST (FDR = 0) and JPX (FDR = 7.44e–64), we find that most of sex-biased lncRNAs (82.5%, 260 out of 315) are expressed in heart. Co-expression analysis further reveals that these lncRNAs are highly correlated (|spearman correlation| > 0.6) with protein-coding transcripts involved in muscle contraction (FDR = 6.2e–18), muscle filament sliding (FDR = 2.8e–10) and sarcomere organization (FDR = 1.1e–2), suggesting their functional roles in cardiac physiology as well as possible contribution to the sex difference in cardiac pathology (76,77). We also identify 132 novel lincRNAs whose expression levels are globally associated with age (FDR < 0.001, Figure 2F and Supplementary Table S12). Among them, the expression levels of 82 novel lincRNAs (62.1%) increase with the elevated age (Figure 2F). In particular, a novel lincRNA, MSTRG.31492.1, is highly expressed in brain and muscle, and its expression levels are positively associated with donor age (FDR = 1.53e–4 globally, 4.79e–4 in brain, and 0.021 in muscle, Figure 2G). The majority of age-associated lncRNAs are transcribed in human brain (97.0%), heart (95.3%) and testis (76.0%). The protein-coding transcripts co-expressed with age-associated lncRNAs (|spearman correlation| > 0.6) are enriched in the biological process of spermatogenesis (FDR = 9.9e–15), cell adhesion (FDR = 5.1e–10), cell differentiation (FDR = 8.6e–10), muscle contraction (FDR = 2.1e–8), multicellular organism development (FDR = 2.3e–4) and chemical synaptic transmission (FDR = 2.9e–3). They are also enriched in the KEGG pathway of focal adhesion (FDR = 1.16e–4), GABAergic synapse (FDR = 1.9e–2), arrhythmogenic right ventricular cardiomyopathy (ARVC) (FDR = 2.0e–2), and hypertrophic cardiomyopathy (HCM) (FDR = 4.0e–2). Furthermore, we detect 70 novel lincRNAs differentially expressed among individuals of various races (FDR < 0.05, Figure 2H and Supplementary Table S13). Notably, compared to novel lincRNAs that are not significantly associated with race, the race-biased ones tend to be expressed in brain (odds ratio [OR] = 8.84, *P*-value = 3.05e–6, Fisher's exact test) and testis (odds ratio [OR] = 2.70, *P*-value = 1.21e–3, Fisher's exact test and see Materials and Methods for details).

Last but not least, we detect 160 novel lincRNAs overlapping with 189 intergenic SNPs reported in 159 genome-wide association studies (51) (Supplementary Table S14). Specifically, 21 novel lincRNAs overlap with cancer-associated SNPs (Supplementary Table S14). For example, the second exon of a novel lincRNA, MSTRG.19068.1, overlaps with a thyroid cancer risk-associated SNP (rs116909374, OR = 1.81, *P*-value = 1e–16; Figure 2I). Interestingly, this novel lincRNA is also specifically expressed in thyroid and significantly up-regulated in thyroid tumor (FDR = 8.84e–38; log_2_FC = 1.39; Figure 2I).

### Novel lincRNAs are dysregulated in various tumors and associated with clinical outcomes

To extend our knowledge beyond the known cancer-associated lncRNAs (78,79) and further explore the potential roles of our newly detected lncRNAs in cancer development, we scan 6,317 tumor samples across 18 tumors in TCGA (Figure 1B). We find 6,674 novel lincRNAs expressed in tumor samples (FPKM > 0.1 in at least one sample), with 734 commonly detected in all 18 tumors (Supplementary Table S15).

**Figure 3.**
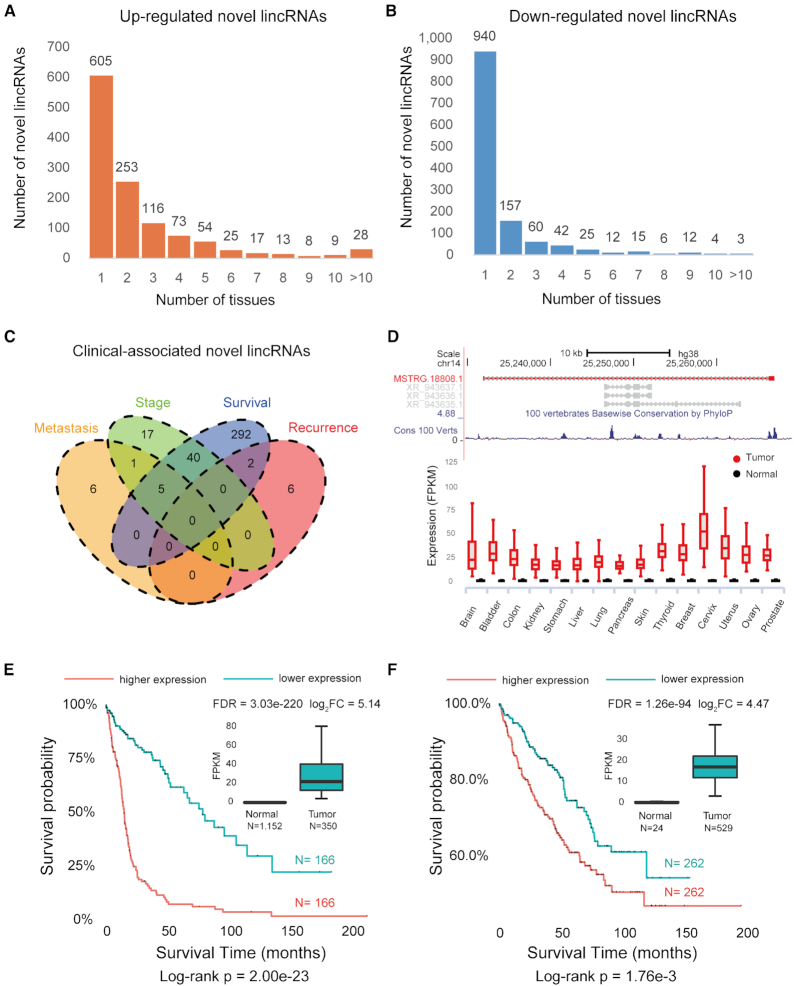
Discovery of tumor-associated novel lincRNAs. (**A**) Novel lincRNAs that are up-regulated in various tumors. (**B**) Novel lincRNAs that are down-regulated in various tumors. (**C**) The Venn diagram of clinical-associated novel lincRNAs. Two novel lincRNAs are both associated with tumor recurrence and patient survival, and five novel lincRNAs are associated with both tumor metastasis and survival. (**D**) The genomic view and differential expression pattern of the survival-associated novel lincRNA MSTRG.18808.1. (**E**) The expression of MSTRG.18808.1 is associated with poorer patient survival in the brain tumor. In the Kaplan-Meier curves of two patient groups with higher (top 50%) or lower (bottom 50%) expression, the red line indicates higher expression, and the blue line indicates lower expression. The box plot in the inset shows that MSTRG.18808.1 has higher expression in the brain tumor (Wilcoxon rank sum test, FDR < 0.05). (**F**) The expression of MSTRG.18808.1 is correlated with poorer patient survival in the kidney tumor. The box plot in the inset shows that MSTRG.18808.1 has higher expression in kidney tumor (Wilcoxon rank sum test, FDR < 0.05).

We further investigate the differential expression patterns of novel lincRNAs in 15 tumors with matched normal tissues available in the GTEx project (see Materials and Methods for details). As a result, 2,163 novel lincRNAs are differentially expressed between tumors and normal tissues, of which 1,201 are up-regulated and 1,276 are down-regulated (Figure 3A, B and Supplementary Table S16). The percentage of tumor-specific RNAs is much higher in the novel lincRNAs (50.4% up- and 73.7% down-regulated, Figure 3A-B) than in previously known lncRNAs (45.3% up- and 50.5% down-regulated, Supplementary Figure S4A and B) or mRNAs (22.4% up- and 37.7% down-regulated, Supplementary Figure S4C and D). Notably, 12 novel lincRNAs show significantly altered expression levels in all 15 tumors and normal counterparts (Supplementary Table S17).

We investigate novel lincRNAs associated with clinical outcomes such as tumor metastasis, recurrence, clinical stage and survival (Figure 3C and see Materials and Methods for details). We identify 12 novel lincRNAs significantly associated with tumor metastasis, and all of them are up-regulated in metastatic tumors (Supplementary Table S18 and Table S19). Additionally, there are eight novel lincRNAs significantly associated with tumor recurrence, and all of them are up-regulated in recurrent tumors (Supplementary Table S18 and Table S20). In addition, 63 novel lincRNAs are significantly associated with clinical stage: 46 are up-regulated in high-stage tumors, and 17 are up-regulated in low-stage tumors (Supplementary Table S18 and Table S21).

To identify the survival-related novel lincRNAs, we perform a multivariate Cox regression analysis for each tumor, controlling for confounding factors such as sex, age and race (see Materials and Methods for details). The expression levels of 339 novel lincRNAs are significantly correlated with overall survival time in nine tumors (FDR < 0.05, Supplementary Table S18 and Table S22). For example, 180 novel lincRNAs are associated with overall survival time in the brain tumor, including 131 putative protective prognostic markers and 49 risky ones. Moreover, about one-half of the survival-associated novel lincRNAs (47.2%, 76/161) are expressed and validated in the independent Chinese LGG dataset (80) of 258 glioma samples with available survival information (FDR < 0.05, Supplementary Table S23). It is also noteworthy that 44 novel lincRNAs show positive or negative correlations with overall survival in at least two tumors, suggesting their potential roles as multiple-tumor prognostic biomarkers (Supplementary Table S24). For example, MSTRG.18808.1 is significantly up-regulated in all 15 tumors (Figure 3D). This novel lincRNA is also correlated with poorer overall survival in the brain tumor (HR = 2.50, FDR = 1.89e–9, Figure 3E, and Supplementary Figure S5A for an independent glioma dataset (HR = 4.81, FDR = 6.37e–9) (80)) and kidney tumor (HR = 2.18, FDR = 2.16e–3, Figure 3F and Supplementary Figure S5B). It is also positively correlated with clinical stage in kidney tumor (FDR = 0.03, Supplementary Figure S5C). In addition to 339 novel intergenic lncRNAs, we also identify 3,525 known lncRNAs with significant association with patient survival time (FDR < 0.05) in at least one tumor (Supplementary Table S22). Nearly one half (1,692 out of 3,525) are also reported as ‘survival-associated lncRNAs’ by TANRIC (81) (Supplementary Table S25). Meanwhile, 207 (89.2%) of 232 survival-associated lncRNAs, which are in the list of disease-associated lncRNAs in the manually curated database EVLncRNA (Experimentally Validated LncRNAs) (82), are curated as ‘cancer-related’ (Supplementary Table S26).

### An interactive web portal

**Figure 4.**
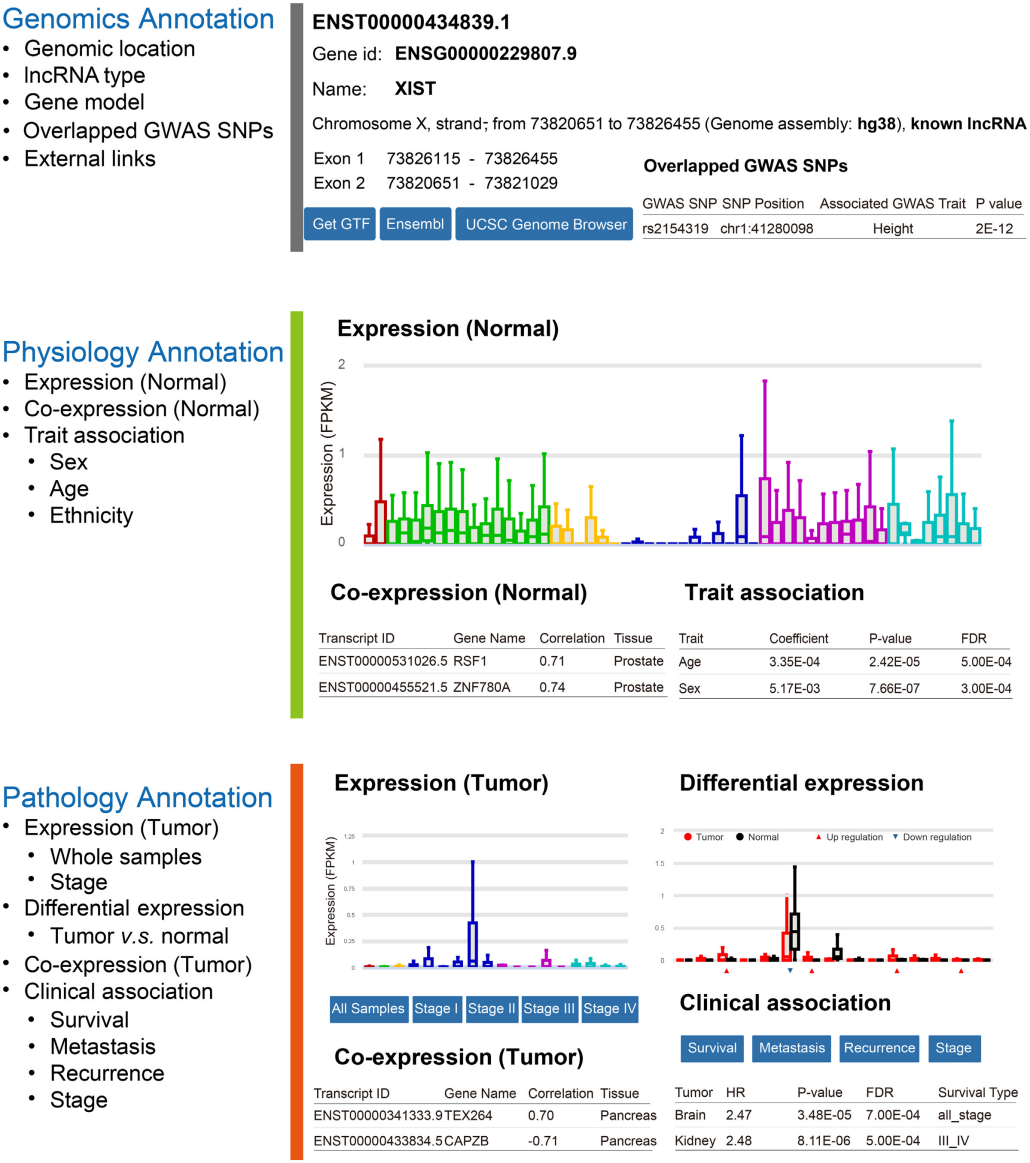
The architecture of the online webserver RefLnc. It provides detailed annotation of each lncRNA in RefLnc including genomics annotation, physiology annotation and pathology annotation.

To facilitate the usage of RefLnc by the wider research community, we develop an online portal for visualizing the detailed characteristics of lncRNAs in 7,849 normal samples and 6,317 tumor samples (Figure 4). This platform allows users to search and download information about the lncRNAs of interest, which is valuable for both experimental and computational researchers. The annotations for each lncRNA are organized into three panels. The *genomics* annotation panel shows the lncRNA’s genomic location, gene model structure and GWAS associations as well as multiple external links to relevant databases. The *physiology* annotation panel displays the lncRNA’s various features in normal samples, including its expression profile, co-expression profile and sex/age/race association results. The *pathology* annotation panel displays the lncRNA’s features in tumor samples, including its expression profile, differential expression profile, co-expression profile and survival association results. In addition, users can obtain more information by using the external link to AnnoLnc, a web server for systematically annotating human lncRNAs (83).

## DISCUSSION

Long noncoding RNAs are emerging as central players in cell biology and play important regulatory roles in various processes such as cell differentiation and development (8,9,78,79). Despite the large number of lncRNAs already reported, the list of human lncRNAs is still far from being completed, partly due to their tissue-specific expression patterns (19). To overcome the challenge, we take a data-driven approach and utilize the largest amount of publicly available data to investigate human lncRNAs. The resultant RefLnc has effectively expanded the landscape of human lncRNAs.

We follow a stringent quality control procedure to remove potential artifacts during transcript assembly and lncRNA identification. 88.5% of novel lncRNAs (95.8% for highly expressed novel lncRNAs) are verified in the independent datasets of 1,131 human normal samples in Sequence Read Archive (SRA) (47) and 935 samples of human cancer cell lines in Cancer Cell Line Encyclopedia (CCLE) (84,85) with >2x per-base coverage on average (67) (Supplementary Figure S6A and B). The transcript quality, measured by the Transcript Confidence Score (TCS), of the novel lncRNAs is remarkably higher than that of known lncRNAs (Figure 1E, *P*-value < 2.2e–16). Compared to known lncRNAs, the novel lncRNAs are less evolutionarily conserved (Figure 2A), have higher expression (Figure 2B) and higher alternative splicing efficiency (Figure 2C). And the novel lncRNAs are expressed in a much more tissue-specific manner than known lncRNAs (Figure 2D). As expected, the novel lncRNAs identified in the present study show remarkably higher inter-individual expression variability than that of known lncRNAs in 23 normal tissues and two cell lines (Supplementary Figure S6C). This high natural expression variability explains why these novel lncRNAs were not identified before.

Among the 27,520 novel lncRNAs, 275 novel lincRNAs are highly correlated with physiological traits of sex, age or race, and 160 novel lincRNAs overlap with intergenic GWAS SNPs. We also identify 2,163 novel lincRNAs differentially expressed between normal and tumor tissues, and 369 novel lincRNAs are associated with clinical outcomes such as patient survival, stage, metastasis and recurrence. Interestingly, compared to uncharacterized novel lincRNAs, these functionally characterized novel lincRNAs show higher conservation across 100 vertebrates (*P*-value = 6.02e–9, Wilcoxon test, Supplementary Figure S6D).

Iyer *et al.* presents a similar large-scale transcriptome survey that *ab initio* assembles 175,706 human lncRNAs (MiTranscriptome, version 2) from 6,503 RNA-Seq samples mainly from tumor (85.8%) (20). Compared to RefLnc, MiTranscriptome has missed 13,414 novel lncRNAs and 12,797 verified known lncRNAs annotated in GENCODE v23, RefSeq and lncRNAdb (Supplementary Figure S7A). Among the RefLnc-specific novel lncRNAs, none of them are extremely lowly expressed (FPKM < 0.1 in all GTEx samples) and 22.5% (3,019/13,414) are of the maximum expression level less than 1 FPKM. Meanwhile, 21.3% (16,668/78,334) of MiTranscriptome-specific lncRNAs are expressed lowly in all of GTEx samples (FPKM < 0.1) and 81.0% (63,472/78,334) are of the maximum expression level less than 1 FPKM. Of note, 17.0% (16,508/97,372) of MiTranscriptome lncRNAs overlapped with RefLnc lncRNAs are also lowly expressed in all of GTEx samples (FPKM < 0.1). Over all, the coverage of MiTranscriptome lncRNAs is significantly lower than that of RefLnc lncRNAs or even novel RefLnc lncRNAs in 14,166 samples in GTEx and TCGA, and independent 2,066 samples of SRA and CCLE (Supplementary Figure S7B–I).

We’ve also performed the comparison of RefLnc with the up-to-date version of FANTOM CAT (56) and CHESS (v2) (86). Compared to RefLnc, FANTOM CAT has missed at least 18,395 novel lncRNAs and 17,928 known lncRNAs annotated in GENCODE, RefSeq and lncRNAdb verified by coverage in 14,166 RNA-Seq samples (Supplementary Figure S8A and S8B). Similarly, CHESS has missed at least 20,313 novel lncRNAs and 14,790 verified known lncRNAs (Supplementary Figure S8C and S8D). Among the 24,172 RefLnc-specific lncRNAs, 16,388 are novel lncRNAs missed by both FANTOM CAT and CHESS (Supplementary Figure S8E and S8F). In which, 15,226 (92.9%) can be verified in the independent datasets of 1,131 human normal samples in SRA (47) and 935 samples of human cancer cell lines in CCLE (84,85) with >2x per-base coverage on average (67). Moreover, among the RefLnc-specific novel lncRNAs, 39 (78%) are validated successfully by independent RT-PCR and Sanger sequencing out of the 50 selected novel lncRNAs.

Since the RefLnc's first release at early 2018, the human lncRNA landscape has been expanded continuously by updated annotations (13–17,20,86–90). The union of the recently updated public lncRNA catalogs (GENCODE v29, RefSeq (NCBI Homo sapiens Annotation Release 109), lncRNAdb v2, NONCODE v5, MiTranscriptome v2, CHESS v2 as well as FANTOM CAT) has covered 16,416 RefLnc novel lncRNAs with >90% coverage, further confirming the high quality of our identification procedure. Meanwhile, there are still 8,842 novel lncRNAs in RefLnc not overlapped with exons of records in any of sources (Supplementary Table S27). Of which, 92.8% (8,209) can be verified in the independent datasets of 1,131 SRA samples and 935 CCLE samples with >2x per-base coverage on average (67). Among the 8,209 verified novel lncRNAs, 16 are correlated with age (FDR < 0.001) and 13 are differentially expressed between two sexes (FDR < 0.05). In addition, 195 are differentially expressed between tumor and normal tissues (FC > 1.5 and FDR < 0.05), 40 are associated with survival time of tumor patient (FDR < 0.05) and 14 are differentially expressed between different clinical stages (FDR < 0.05). This well highlights the great necessity for improving lncRNA annotations in term of completeness and comprehensiveness (91,92).

It should be noted that our analysis is restricted to poly-A+ transcripts and offer little insight into expression of lncRNAs that lack poly(A) tails. Therefore, more sophisticated methods, such as non-poly(A) tail RNA-seq technology, are required to more comprehensively capture the lncRNA transcriptome.

Overall, RefLnc has greatly expanded the landscape of human lncRNAs and enabled the genome-wide exploration of the physiological function and clinical significance of lncRNAs. We anticipate that the RefLnc assembly as well as the computational pipelines developed will help to advance our knowledge of lncRNAs and provide a foundation for lncRNA genomics and biomarker development.

## DATA AVAILABILITY

The processed data and all code for functional analysis are available in the RefLnc online webserver (http://reflnc.gao-lab.org/).

## Supplementary Material

gkz621_Supplemental_Files
