# Supplementary material for: An expanded landscape of human long noncoding RNA
Source: Nucleic Acids Res. 2019 Jul 27;47(15):7842–56. doi: 10.1093/nar/gkz621 (PMC6735957; doi:10.1093/nar/gkz621)
Supplement: gkz621_Supplemental_Files [file gkz621_supplemental_files.zip › Supplementary Figures.pdf]

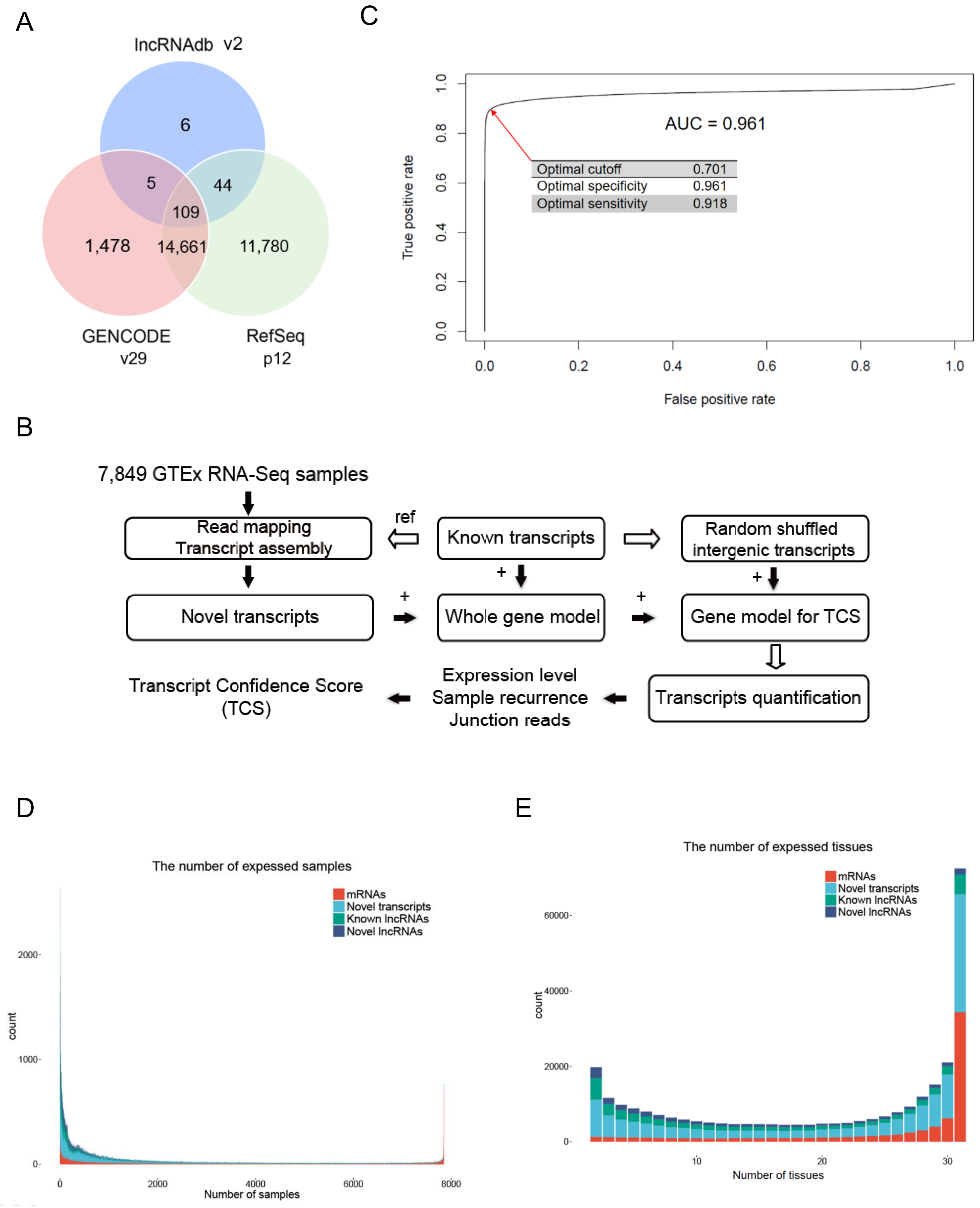

A
lncRNAdb v2
6
5
44
109
1,478
14,661
11,780
GENCODE v29
RefSeq p12
C
AUC = 0.961
Optimal cutoff 0.701
Optimal specificity 0.961
Optimal sensitivity 0.918
True positive rate
False positive rate
0.0
0.2
0.4
0.6
0.8
1.0
B
7,849 GTEx RNA-Seq samples
Read mapping
Transcript assembly
ref
Known transcripts
Random shuffled intergenic transcripts
Novel transcripts
Whole gene model
Gene model for TCS
Transcript Confidence Score (TCS)
Expression level
Sample recurrence
Junction reads
Transcripts quantification
D
The number of expressed samples
mRNAs
Novel transcripts
Known lncRNAs
Novel lncRNAs
count
2000
1000
0
0
2000
4000
6000
8000
Number of samples
E
The number of expressed tissues
mRNAs
Novel transcripts
Known lncRNAs
Novel lncRNAs
count
60000
40000
20000
0
10
20
30
Number of tissues

F

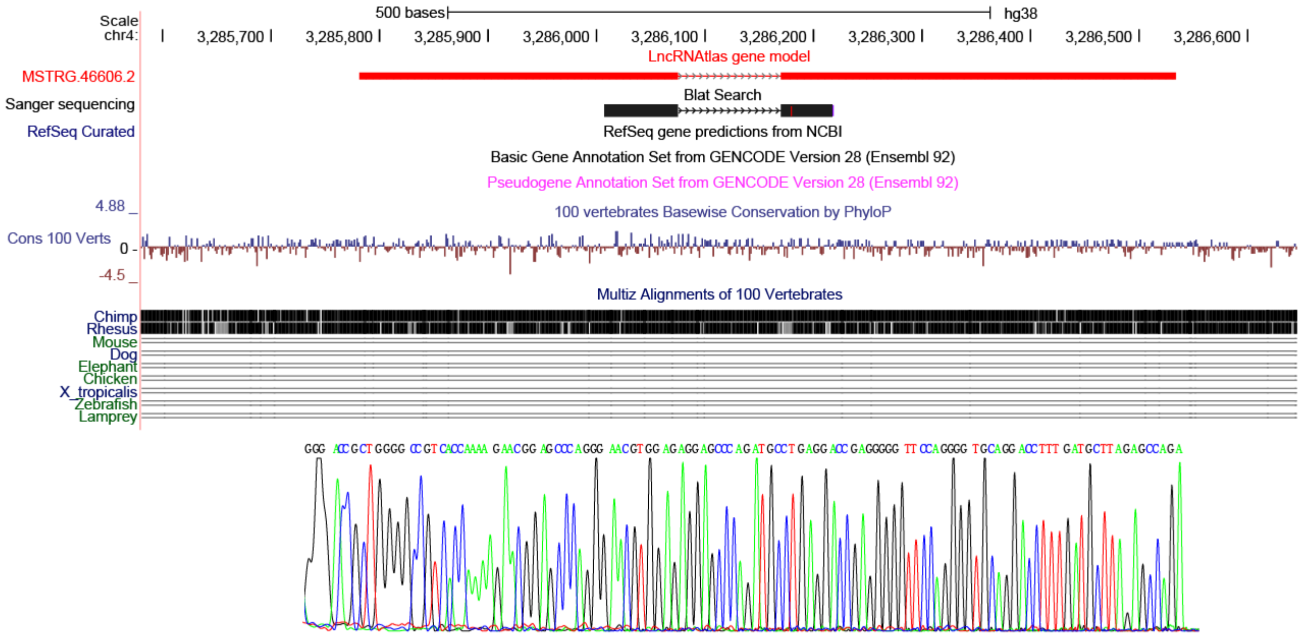
Scale
chr4:
500 bases
hg38
3,285,700
3,285,800
3,285,900
3,286,000
3,286,100
3,286,200
3,286,300
3,286,400
3,286,500
3,286,600
LncRNAtlas gene model
MSTRG.46606.2
Blat Search
Sanger sequencing
RefSeq Curated
RefSeq gene predictions from NCBI
Basic Gene Annotation Set from GENCODE Version 28 (Ensembl 92)
Pseudogene Annotation Set from GENCODE Version 28 (Ensembl 92)
4.88
100 vertebrates Basewise Conservation by PhyloP
Cons 100 Verts
0
-4.5
Multiz Alignments of 100 Vertebrates
Chimp
Rhesus
Mouse
Dog
Elephant
Chicken
X_tropicalis
Zebrafish
Lamprey

G

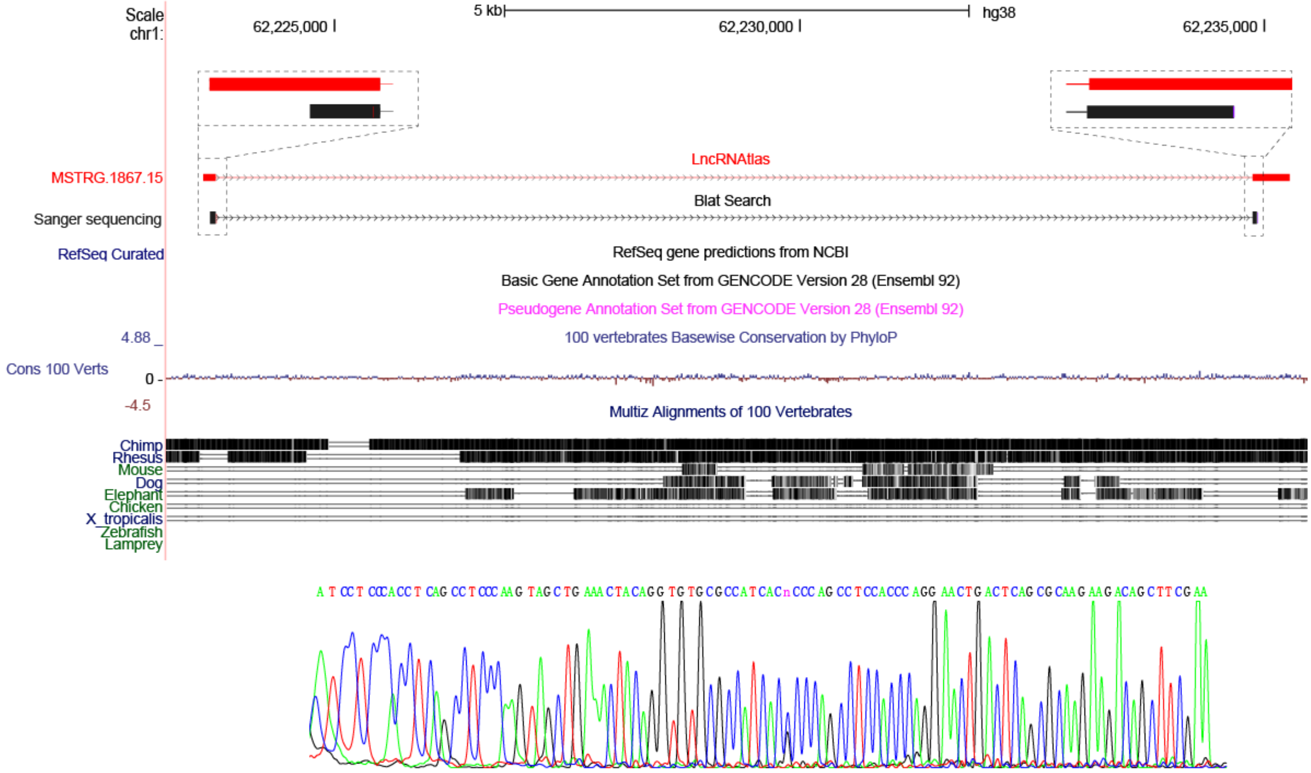
Scale
chr1:
5 kb
hg38
62,225,000
62,230,000
62,235,000
LncRNAtlas
MSTRG.1867.15
Blat Search
Sanger sequencing
RefSeq Curated
RefSeq gene predictions from NCBI
Basic Gene Annotation Set from GENCODE Version 28 (Ensembl 92)
Pseudogene Annotation Set from GENCODE Version 28 (Ensembl 92)
4.88
100 vertebrates Basewise Conservation by PhyloP
Cons 100 Verts
0
-4.5
Multiz Alignments of 100 Vertebrates
Chimp
Rhesus
Mouse
Dog
Elephant
Chicken
X_tropicalis
Zebrafish
Lamprey

H

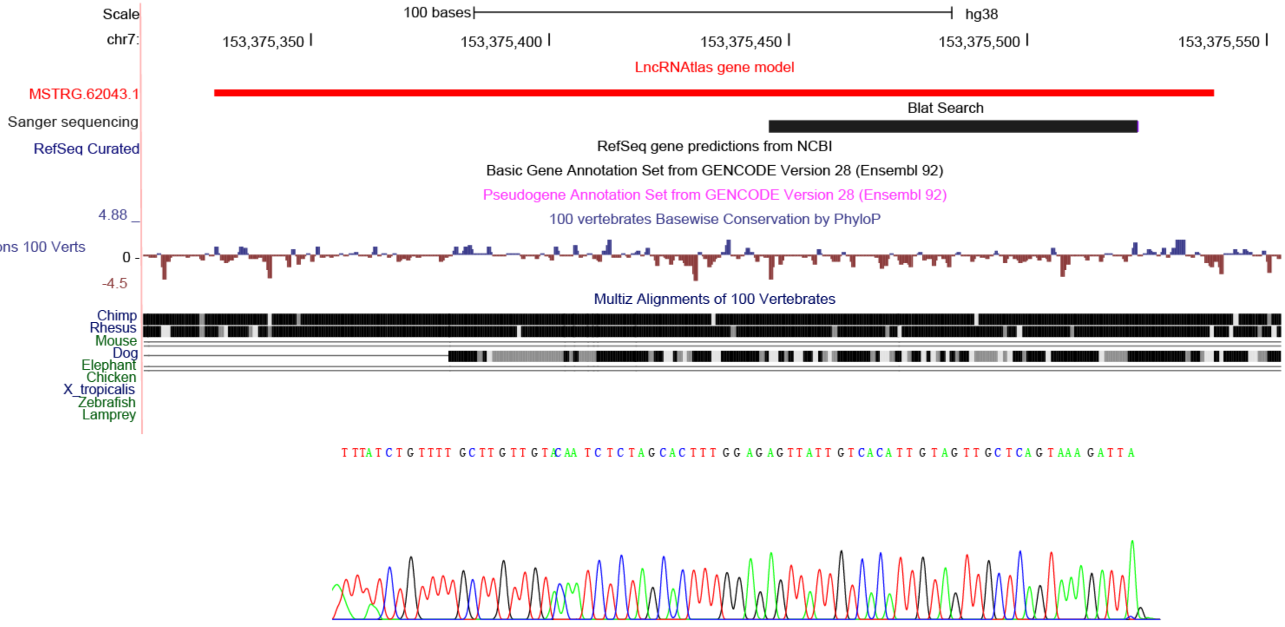

I

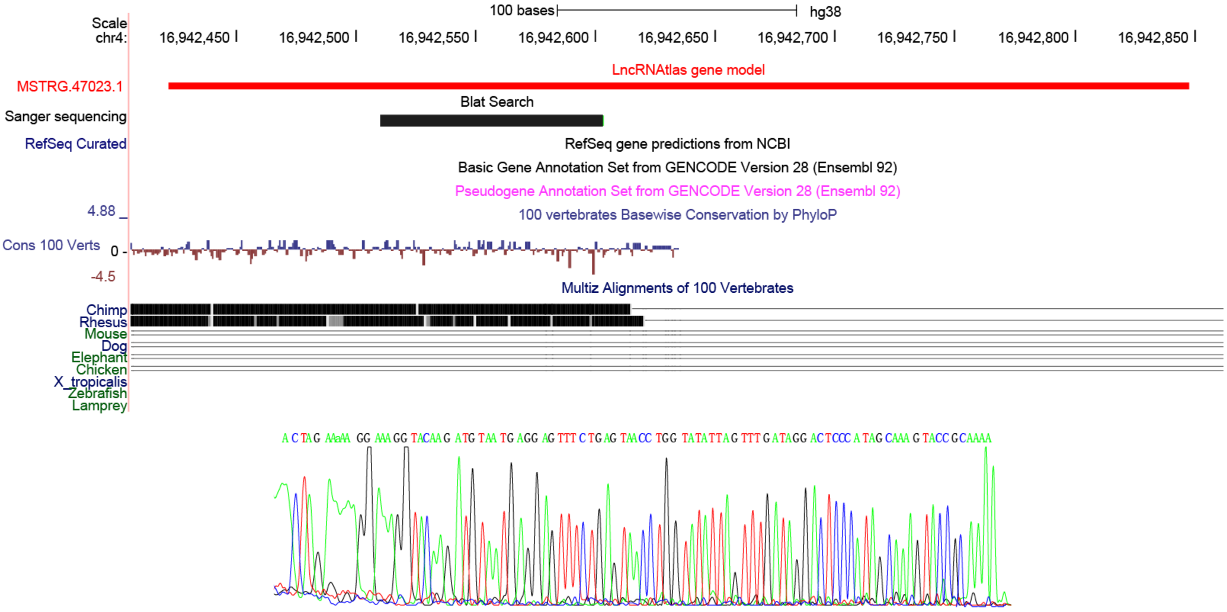

Supplementary Figure S1. The comparison of lncRNA reference catalogs and transcript quality evaluation of novel lncRNAs. (A) 23.4% (13,264/56,804) of lncRNAs are found in only one lncRNA catalog among GENCODE v29, RefSeq (NCBI Homo sapiens Annotation Release 109) and lncRNAdb v2. The overlap of 1 bp in the transcript locus (including introns, ignoring strand) is considered as redundant. Given the internal redundancy of each catalog, the overlap is the minimal number of overlaps between various catalogs in order not to produce negative area when drawing the Venn diagram. The number of unique genes in each catalog shown in this Venn diagram is the exact number. **(B)** For the ROC analysis of TCS, we generate random intergenic transcripts according to the structures of known mRNAs and lncRNAs. Then we merge these random intergenic transcripts with the whole gene model, and calculate the expression level and other features needed in TCS calculation for the merged gene model. By this method, we calculate the TCS for the randomly shuffled intergenic transcripts and perform the ROC analysis. **(C)** The Transcript Confident Score (TCS) shows good performance, with AUC of 0.961 and high specificity (0.961) and sensitivity (0.918), under the optimal cutoff. **(D)** The number of tissues in which

transcripts are expressed (>0.1 FPKM). **(E)** The number of samples in which transcripts are expressed (>0.1 FPKM). **(F-G)** Example of two novel intergenic multi-exon lncRNAs that are validated by Sanger sequencing. The blat position of Sanger sequencing result fully matches the junction of novel lncRNAs. **(H-I)** Example of two novel intergenic single-exon lncRNAs that are validated by Sanger sequencing.

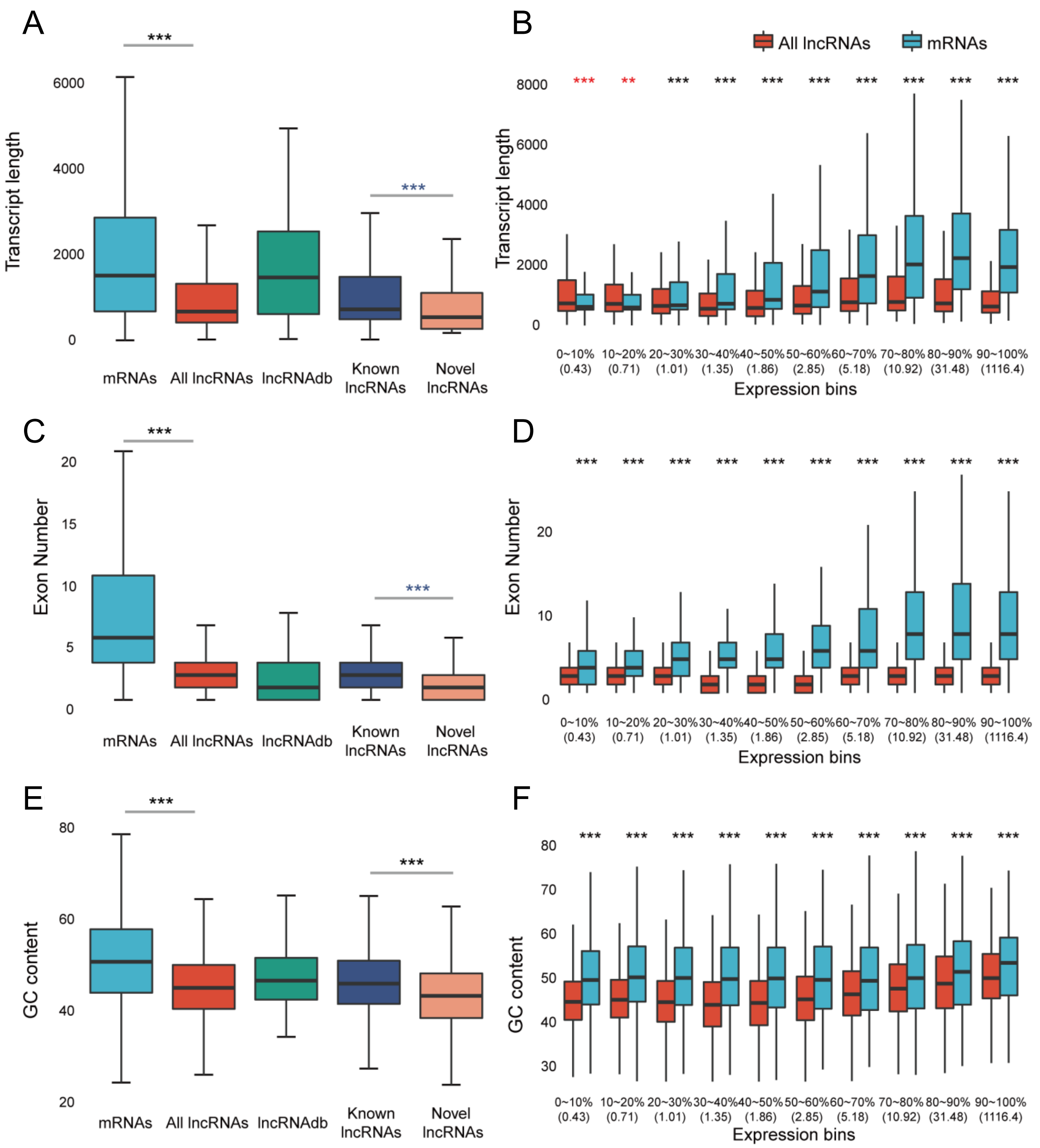

Supplementary Figure S2. The characterization of sequence features for lncRNAs and mRNAs. (A) lncRNAs have shorter transcript lengths than those of mRNAs, and novel lncRNAs have shorter transcript lengths than those of known lncRNAs. **(B)** lncRNAs have shorter transcript length than those of mRNAs with similar expression levels. **(C)** lncRNAs have fewer exons than mRNAs, and novel lncRNAs have fewer exons than known lncRNAs. **(D)** lncRNAs have fewer exons than mRNAs with similar expression levels. **(E)** lncRNAs have lower GC content than that of mRNAs, and novel lncRNAs have lower GC content than that of known lncRNAs. **(F)** lncRNAs have lower GC content than that of mRNAs with similar expression levels. In panels b, d and f, the expression level is categorized into ten bins according to expression rank (maximum expression across all physiological samples) for all lncRNAs and mRNAs. The number in brackets represents the maximum expression (FPKM) in each bin. In panels b, d and f, the red asterisks represent lncRNAs that have a larger value than that of the mRNAs, while the black asterisks indicate mRNAs that have a larger value than lncRNAs. P-values are calculated using the Wilcoxon rank sum test. “*”: 0.01 < p-value < 0.05; “**”: 0.001 < p-value < 0.01; “***”: p-value < 0.001.

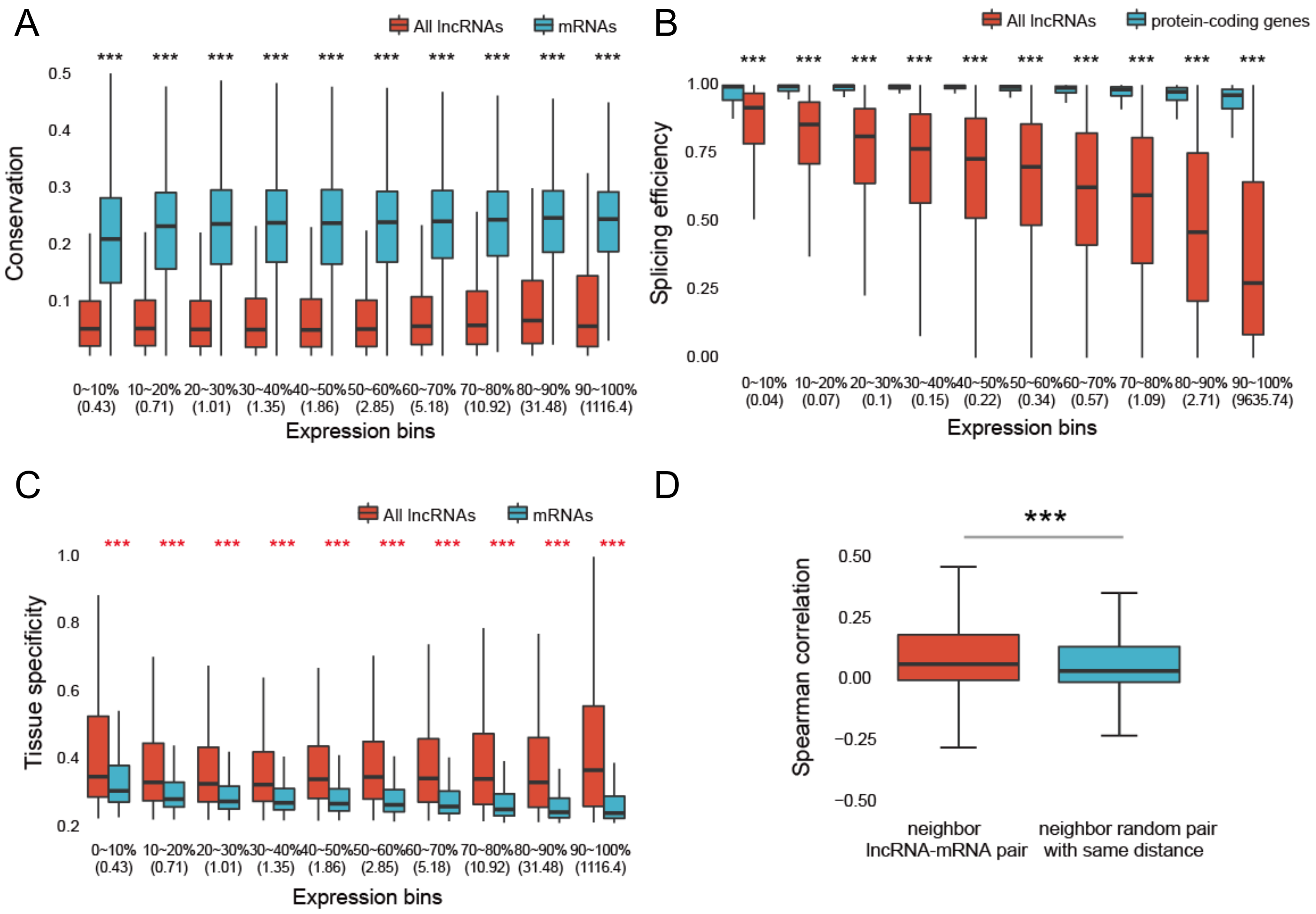

Supplementary Figure S3. The characterization of conservation and expression features for lncRNAs and mRNAs. (A) lncRNAs are less conserved than mRNAs with similar expression levels. **(B)** lncRNAs have lower splicing efficiency than mRNAs with similar expression levels. **(C)** lncRNAs are expressed in a much more tissue-specific manner than mRNAs with similar expression levels. The expression level is categorized into ten bins according to expression rank (maximum expression across all physiological samples) for all lncRNAs and mRNAs. **(D)** The Spearman correlation of lncRNA-mRNA neighbor pairs is significantly higher than the correlation of random neighbor pairs with the same distance. The random pairs are selected randomly from neighboring pairs of transcripts other than lncRNAs and mRNAs, and the random pairs are of the same distance with lncRNA-mRNA neighbor pairs. P-values are calculated using the Wilcoxon rank sum test. "*": 0.01 < p-value < 0.05; "**": 0.001 < p-value < 0.01; "***": p-value < 0.001.

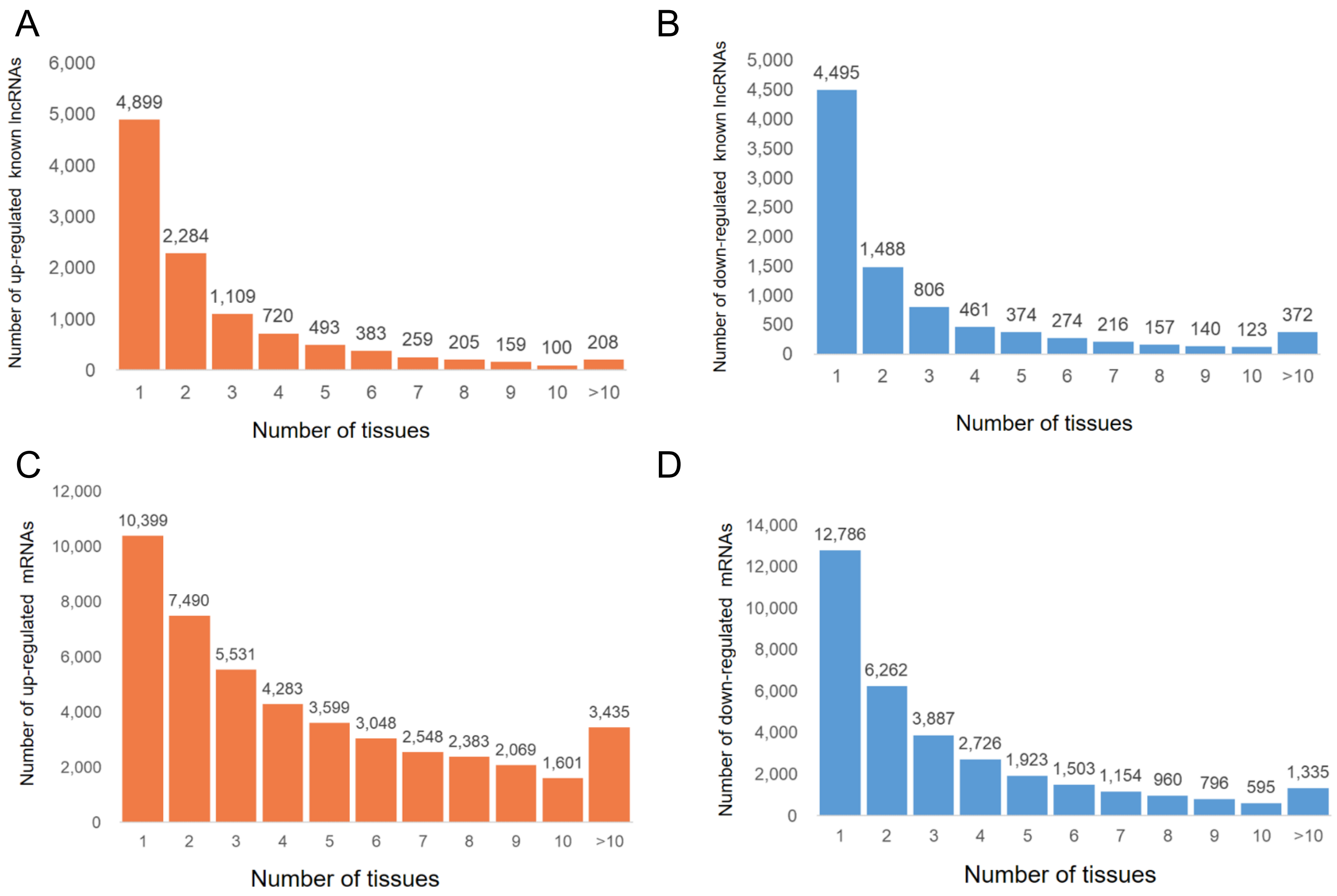

Supplementary Figure S4. Analysis of differentially expressed lncRNAs and mRNAs between tumor and normal tissues. (A) The number of known lncRNAs up-regulated in various tumors. **(B)** The number of known lncRNAs downregulated in various tumors. **(C)** The number of mRNAs up-regulated in various tumors. **(D)** The number of mRNAs down-regulated in various tumors.

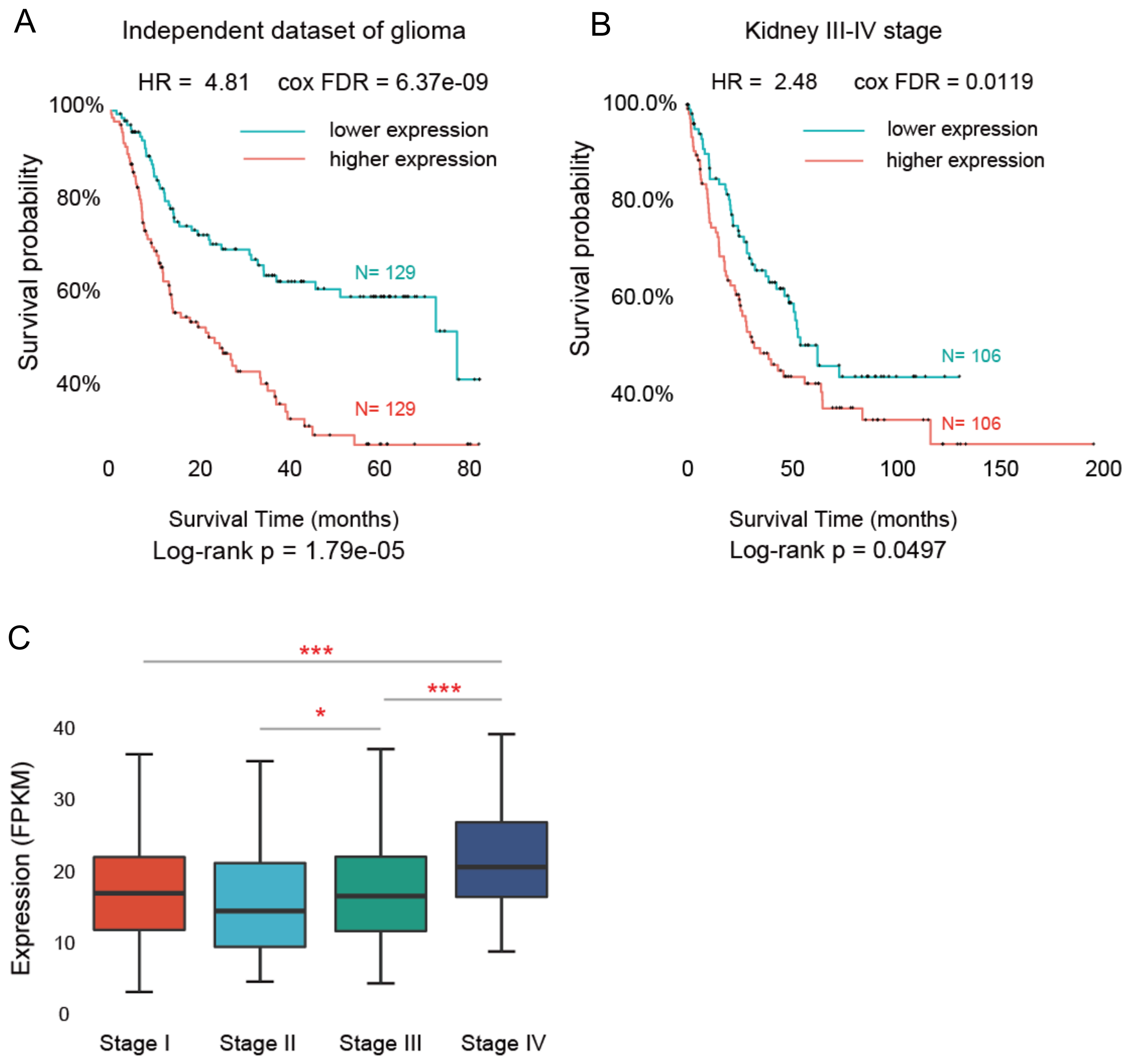

Supplementary Figure S5. The clinical association of novel lincRNA MSTRG.18808.1. (A) The expression of the novel lincRNA MSTRG.18808.1 is associated with poorer patient survival in the independent Chinese LGG dataset, which includes 258 glioma samples with available survival information. **(B)** The expression of the novel lincRNA MSTRG.18808.1 is correlated with poorer patient survival in kidney tumor of stages III to IV. **(C)** Higher expression of MSTRG.18808.1 is positively correlated with higher clinical stage in kidney tumor.

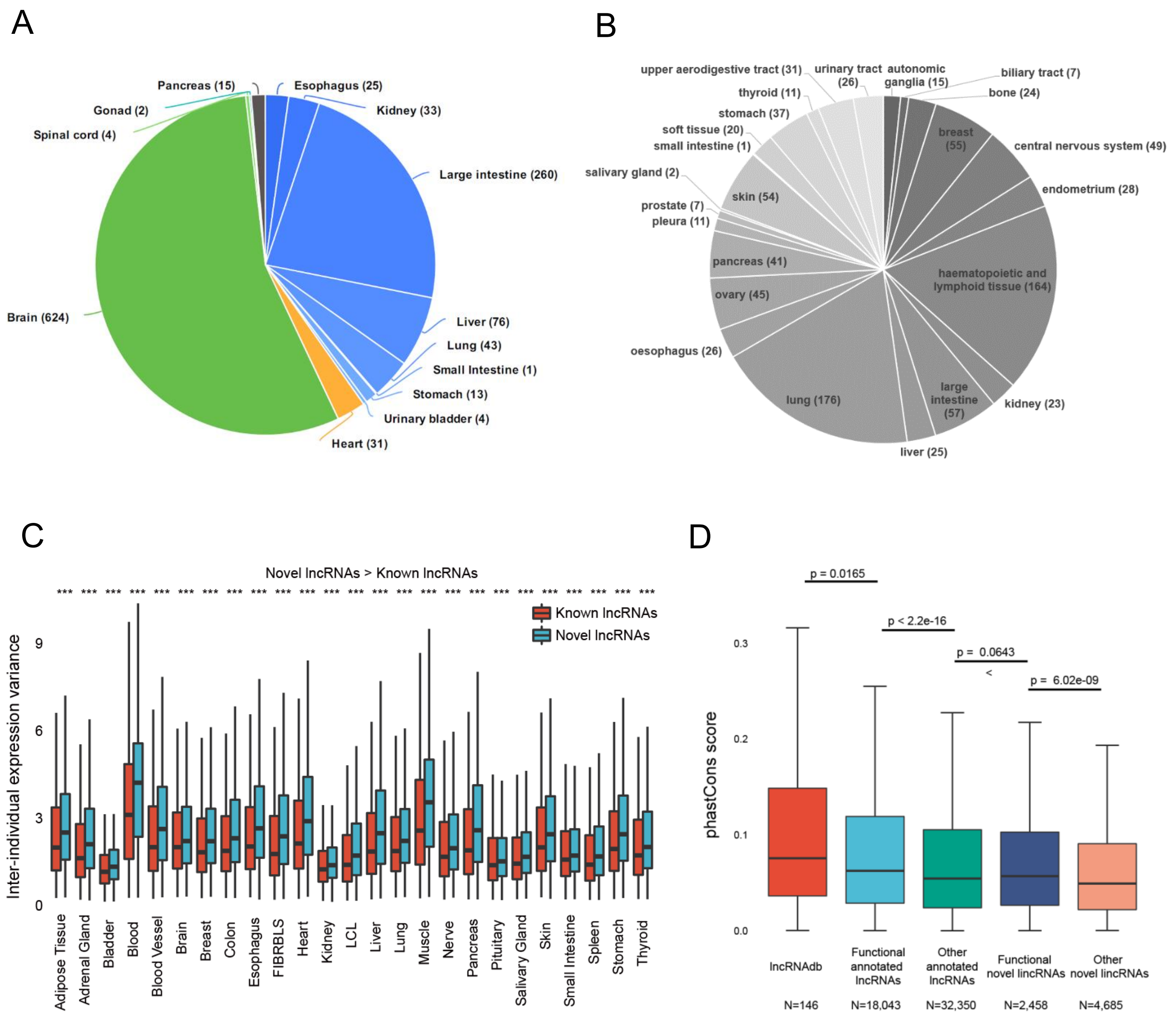

Supplementary Figure S6. The independent datasets for analysis and the inter-individual expression variance and conservation of novel lncRNAs. (A) The composition of the 1,131 independent human normal samples from the SRA. **(B)** The composition of the 935 independent human cancer cell line samples from the CCLE. **(C)** Novel lncRNAs have higher inter-individual expression variability than that of known lncRNAs in all 25 tissues. P values are calculated using the Wilcoxon rank sum test. “*”: 0.01 < p-value < 0.05; “**”: 0.001 < p-value < 0.01; “***”: p-value < 0.001. **(D)** The putatively novel functional lincRNAs show higher conservation across 100 vertebrates than other non-characterized novel lincRNAs. The putatively functional novel lincRNAs are transcripts associated with any of the following traits: (i) overlapped with significant trait-associated SNPs located within intergenic regions; (ii) differentially expressed between tumor and normal samples; (iii) significantly correlated with physiological traits (sex/age/ethnicity); (iv) associated with cancer survival, metastasis, clinical stage or recurrence.

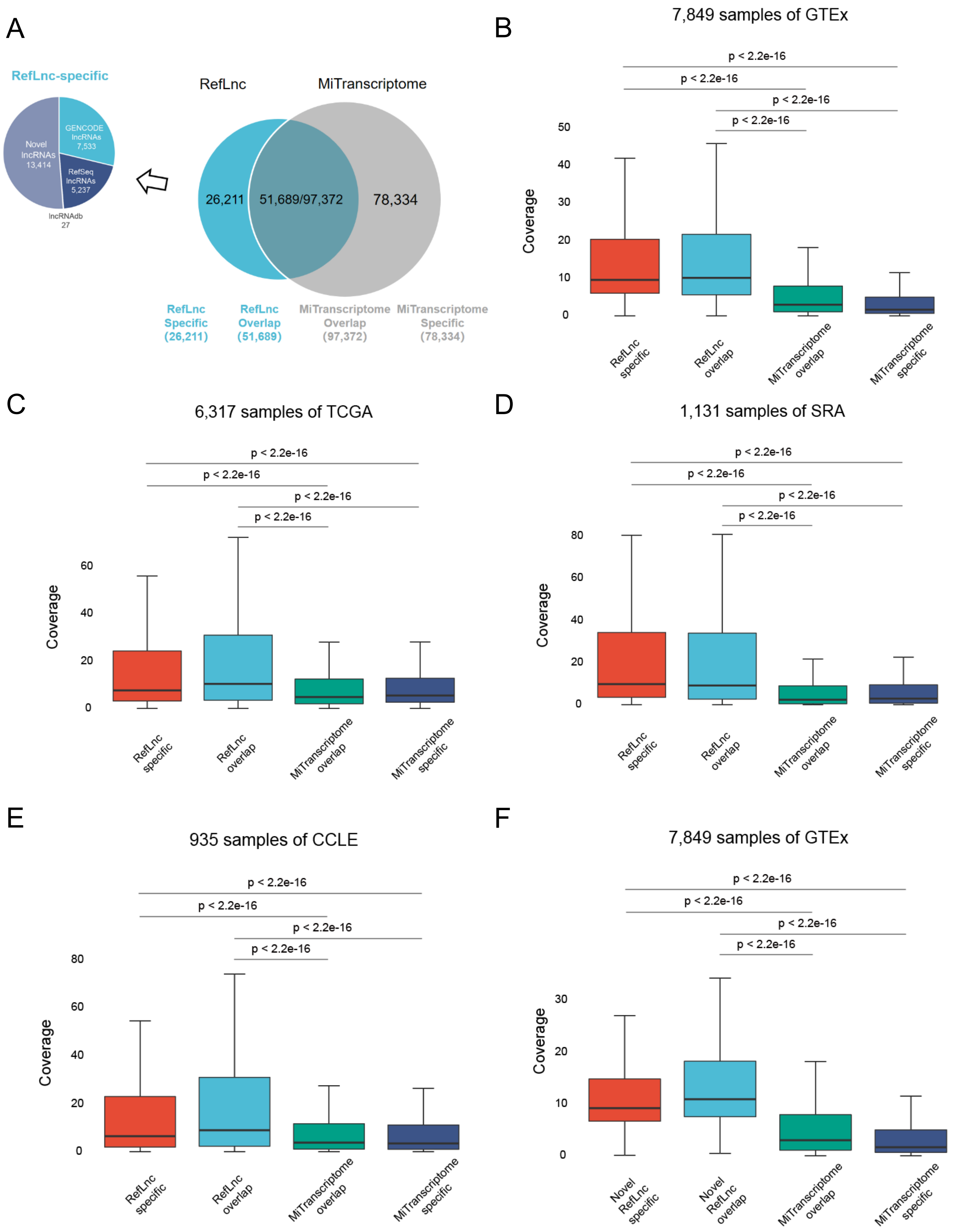
A
RefLnc-specific
GENCODE lncRNAs 7,533
RefSeq lncRNAs 5,237
lncRNAdb 27
Novel lncRNAs 13,414
RefLnc
MiTranscriptome
26,211
51,689/97,372
78,334
RefLnc Specific (26,211)
RefLnc Overlap (51,689)
MiTranscriptome Overlap (97,372)
MiTranscriptome Specific (78,334)
B
7,849 samples of GTEx
p < 2.2e-16
Coverage
RefLnc specific
RefLnc overlap
MiTranscriptome overlap
MiTranscriptome specific
C
6,317 samples of TCGA
D
1,131 samples of SRA
E
935 samples of CCLE
F
7,849 samples of GTEx
Novel RefLnc specific
Novel RefLnc overlap

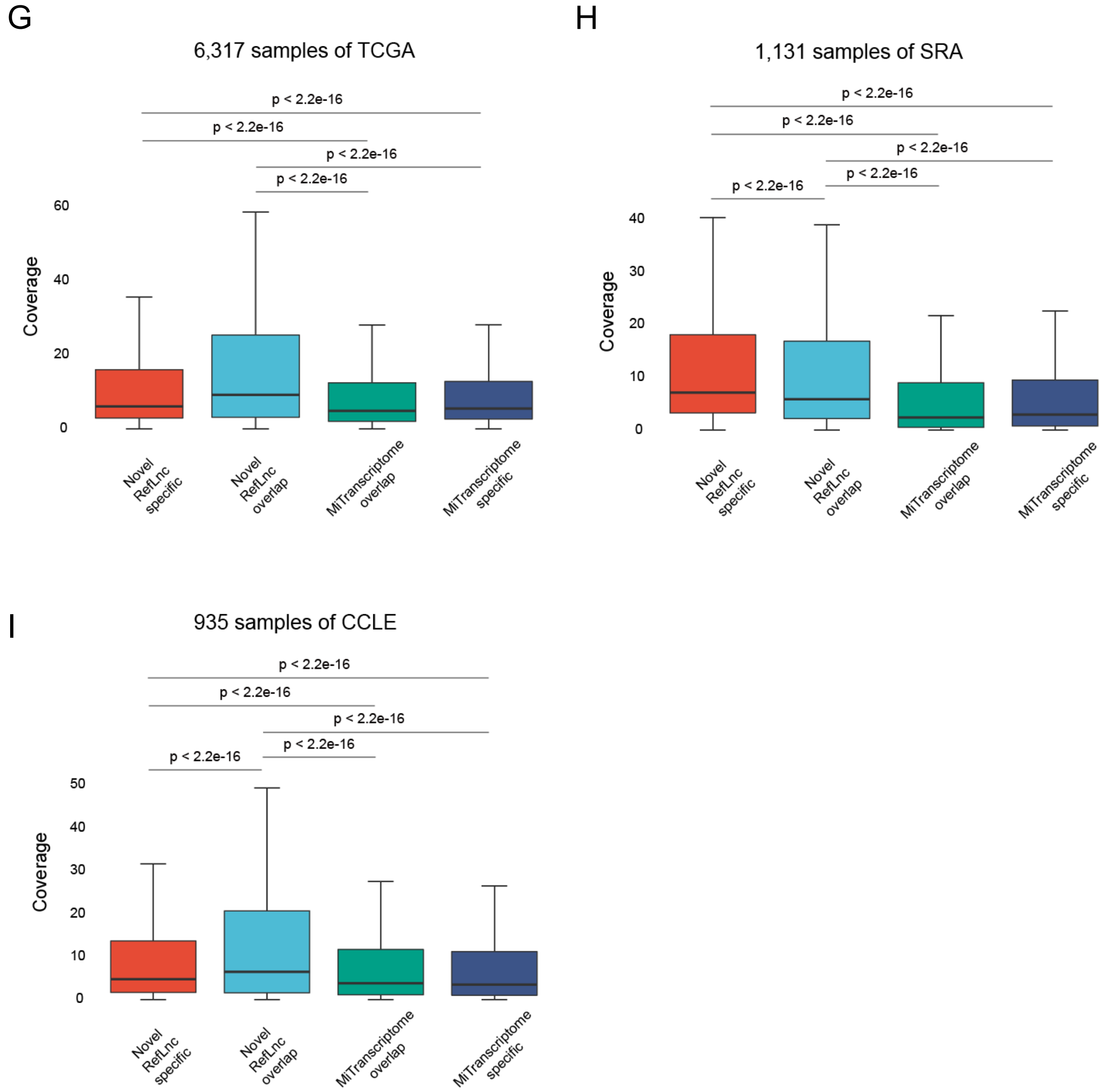

Supplementary Figure S7. The comparison between RefLnc and MiTranscriptome and coverage distribution in 14,166 samples in GTEx and TCGA, and independent 2,066 samples of SRA and CCLE. (A) RefLnc overlaps 55.4% of MiTranscriptome lncRNAs, and MiTranscriptome has missed 13,414 novel lncRNAs and 12,797 verified known lncRNAs annotated in GENCODE v23, RefSeq and lncRNAdb. The overlap of 1 bp in the exon (ignoring strand) is considered as redundant. All of lncRNAs of RefLnc have significant higher coverage (maximum coverage) than lncRNAs of MiTranscriptome in **(B)** 7,849 GTEx samples, **(C)** 6,317 TCGA samples, **(D)** 1,131 SRA samples or **(E)** 935 CCLE samples. The novel of lncRNAs of RefLnc have higher coverage than lncRNAs of MiTranscriptome in **(F)** 7,849 GTEx samples, **(G)** 6,317 TCGA samples, **(H)** 1,131 SRA samples or **(I)** 935 CCLE samples.

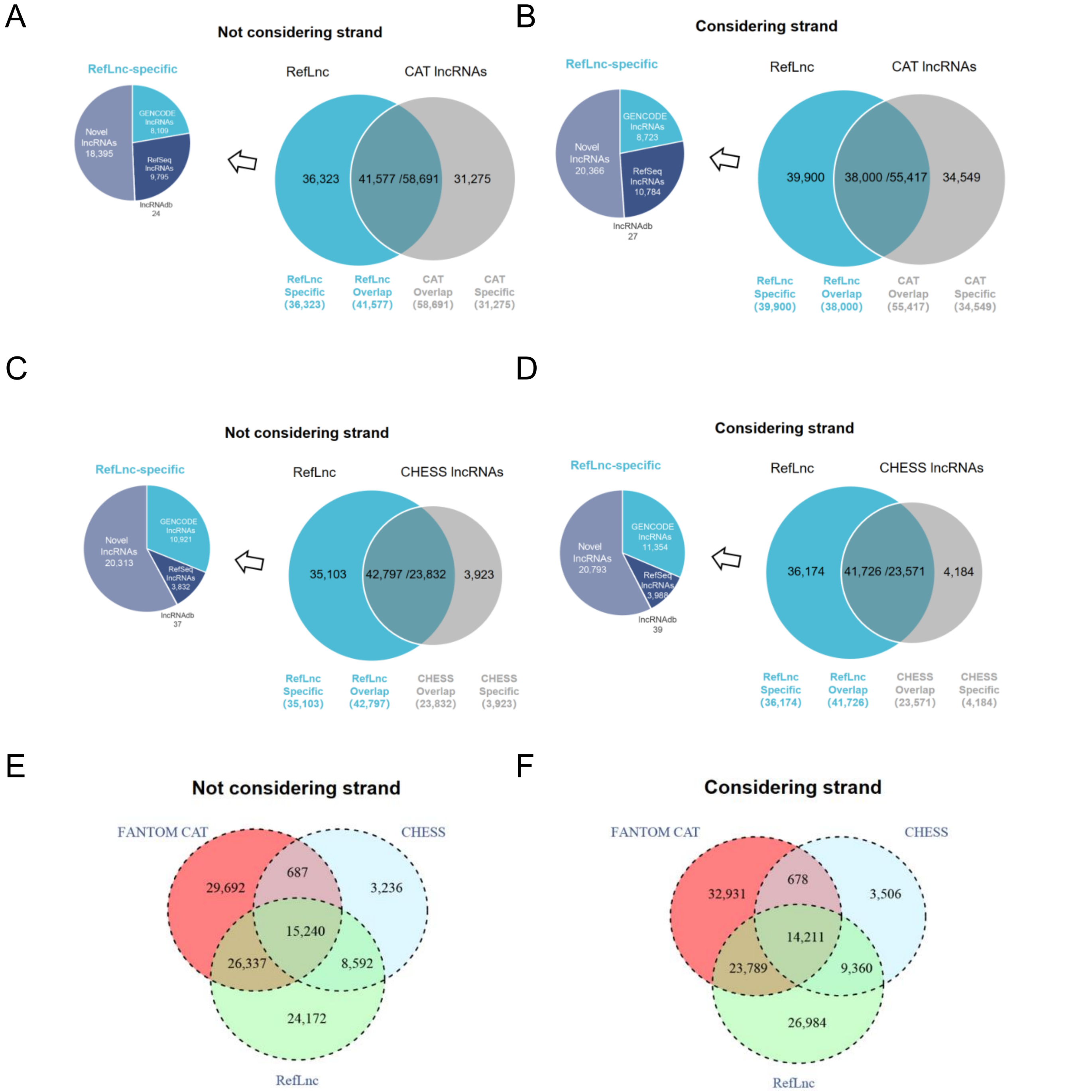

Supplementary Figure S8. The comparison between RefLnc and FANTOM CAT/CHESS. (A) Compared to RefLnc (the overlap of 1 bp in the exon regardless of strand is considered as redundant), FANTOM CAT has missed 18,395 novel lncRNAs and 17,928 known lncRNAs annotated in GENCODE, RefSeq and lncRNAdb verified by coverage in 14,166 RNA-Seq samples. Among the 18,395 RefLnc-specific novel lncRNAs, 91.1% (16,753) can be verified in the independent datasets of 1,131 human normal samples in SRA (1) and 935 samples of human cancer cell lines in CCLE (2,3) with >2x per-base coverage on average (4). Moreover, among the 18,395 RefLnc-specific novel lncRNAs, 42 (79.2%) are validated successfully out of 53 selected cases. **(B)** The comparison between RefLnc and FANTOM CAT (the overlap of 1 bp in the exon with the same strand is considered as redundant). **(C)** CHESS has missed 20,313 novel lncRNAs and 14,790 verified known lncRNAs annotated in GENCODE, RefSeq and lncRNAdb (the overlap of 1 bp in the exon regardless of strand is considered as

redundant). Among the 20,313 RefLnc-specific novel lncRNAs missed by CHESS, 92.7% (18,825) can be verified in the independent datasets of 1,131 human normal samples in SRA (1) and 935 samples of human cancer cell lines in CCLE (2,3) with >2x per-base coverage on average (4). Moreover, among the RefLnc-specific novel lncRNAs, 62 (80.5%) are validated successfully out of 77 selected cases. **(D)** The comparison between RefLnc and CHESS (the overlap of 1 bp in the exon with the same strand is considered as redundant). **(E)** Among the 24,172 RefLnc-specific lncRNAs (the overlap of 1 bp in the exon regardless of strand is considered as redundant), 16,388 are novel lncRNAs missed by both FANTOM CAT and CHESS. **(F)** The comparison between RefLnc, CAT and CHESS (the overlap of 1 bp in the exon with the same strand is considered as redundant). Given the internal redundancy of each catalog, the overlap is the minimal number of overlaps between various catalogs in order not to produce negative area when drawing the Venn diagram. The number of unique genes in each catalog shown in this Venn diagram is the exact number.

1. Leinonen, R., Sugawara, H., Shumway, M. and International Nucleotide Sequence Database, C. (2011) The sequence read archive. *Nucleic acids research*, **39**, D19-21.
2. Barretina, J., Caponigro, G., Stransky, N., Venkatesan, K., Margolin, A.A., Kim, S., Wilson, C.J., Lehar, J., Kryukov, G.V., Sonkin, D. *et al.* (2012) The Cancer Cell Line Encyclopedia enables predictive modelling of anticancer drug sensitivity. *Nature*, **483**, 603-607.
3. Cancer Cell Line Encyclopedia, C. and Genomics of Drug Sensitivity in Cancer, C. (2015) Pharmacogenomic agreement between two cancer cell line data sets. *Nature*, **528**, 84-87.
4. Li, S., Labaj, P.P., Zumbo, P., Sykacek, P., Shi, W., Shi, L., Phan, J., Wu, P.Y., Wang, M., Wang, C. *et al.* (2014) Detecting and correcting systematic variation in large-scale RNA sequencing data. *Nature biotechnology*, **32**, 888-895.
